# Significance of chitinase-3-like protein 1 in the pathogenesis of inflammatory diseases and cancer

**DOI:** 10.1038/s12276-023-01131-9

**Published:** 2024-01-04

**Authors:** Ji Eun Yu, In Jun Yeo, Sang-Bae Han, Jaesuk Yun, Bongcheol Kim, Yoon Ji Yong, Young-soo Lim, Tae Hun Kim, Dong Ju Son, Jin Tae Hong

**Affiliations:** 1 College of Pharmacy and Medical Research Center, Chungbuk National University, 194-31, Osongsaengmyeong 1-ro, Osong-eup, Cheongju-si, Chungbuk 28160 Republic of Korea; 2 College of Pharmacy, Kyungpook National University, 80 Daehakro, Bukgu, Daegu, 41566 Republic of Korea; 3 Senelix Co. Ltd., 25, Beobwon-ro 11-gil, Songpa-gu, Seoul, 05836 Republic of Korea; 4 PRESTI GEBIOLOGICS Co. Ltd., Osongsaengmyeong 1-ro, Osong-eup, Cheongju-si, Chungbuk 28161 Republic of Korea; 5 Autotelic Bio Inc., Osongsaengmyeong 1-ro, Osong-eup, Heungdeok-gu, Cheongju-si, Chungbuk 28160 Republic of Korea

**Keywords:** Predictive markers, Tumour biomarkers, Atherosclerosis, Alzheimer's disease

## Abstract

Chitinase-3-like protein 1 (CHI3L1) is a secreted glycoprotein that mediates inflammation, macrophage polarization, apoptosis, and carcinogenesis. The expression of CHI3L1 is strongly upregulated by various inflammatory and immunological diseases, including several cancers, Alzheimer’s disease, and atherosclerosis. Several studies have shown that CHI3L1 can be considered as a marker of disease diagnosis, prognosis, disease activity, and severity. In addition, the proinflammatory action of CHI3L1 may be mediated via responses to various proinflammatory cytokines, including tumor necrosis factor-α, interleukin-1β, interleukin-6, and interferon-γ. Therefore, CHI3L1 may contribute to a vast array of inflammatory diseases. However, its pathophysiological and pharmacological roles in the development of inflammatory diseases remain unclear. In this article, we review recent findings regarding the roles of CHI3L1 in the development of inflammatory diseases and suggest therapeutic approaches that target CHI3L1.

## Protein CHI3L1: a potential game-changer in inflammatory disease treatment

Chitinase-3-like protein 1 (CHI3L1) is a secreted glycoprotein with diverse roles in inflammation, macrophage polarization, apoptosis, and carcinogenesis. Pro-inflammatory effects of CHI3L1 are attributed to its response to various pro-inflammatory cytokines, including tumor necrosis factor-α, interleukin-1β, interleukin-6, and interferon-γ. Consequently, CHI3L1 is implicated in a wide range of inflammatory diseases. This review summarizes the significance of CHI3L1 as a potential target in multiple inflammatory diseases and cancer by analyzing data from platforms like Open Targets and other data-analysis tools. Furthermore, we have utilized platforms like STRING to identify potential target proteins for CHI3L1 in various inflammatory diseases and cancer. In this article, we provide a comprehensive review of recent findings regarding the involvement of CHI3L1 in the development of inflammatory diseases and cancer. Finally, we propose therapeutic approaches targeting CHI3L1 for treatment of these diseases. This summary was initially drafted using artificial intelligence, then revised and fact-checked by the author.

## Introduction

Although several studies on chitinase-3-like 1 (CHI3L1) have been published, a systematic review of the various features and functions of this protein is lacking. In this review article, we provide information based on an analysis of the available data using an Open Targets Platform^1–3^ and other data-analysis platforms to assess the significance of CHI3L1 as a target molecule in several inflammatory diseases. We also provide further information regarding the interacting target of CHI3L1 obtained using the Search Tool for the Retrieval of Interacting Genes/Proteins (STRING), which is a comprehensive web-based platform that lists known and predicted protein–protein interactions^4–9^. Furthermore, we discuss recent findings related to the roles of CHI3L1 in the development of several inflammatory diseases, as well as in therapeutic approaches.

**Fig. 1:**
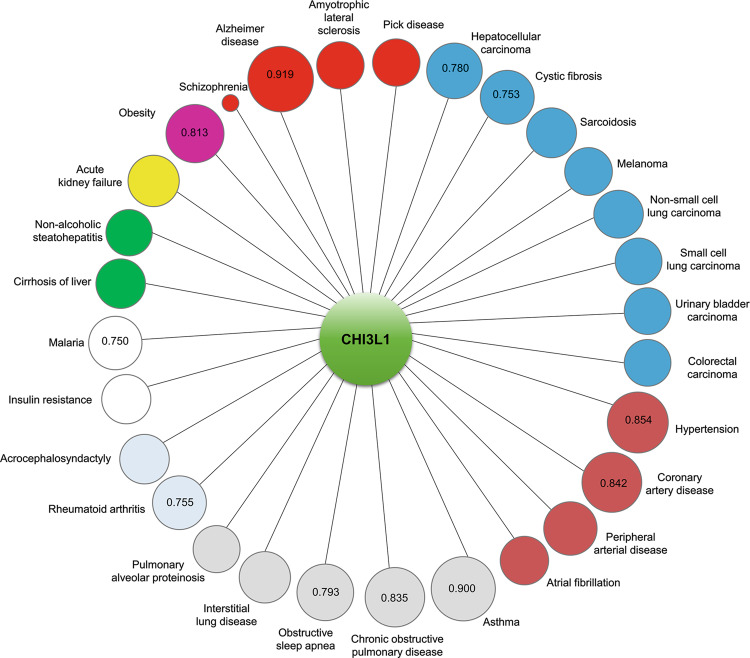
Relationship between CHI3L1 and various diseases. The circle sizes are determined based on text-mining scores. The values in the circle symbols are the text-mining scores determined via Open Targets Platform analysis.

Using the Open Targets Platform, we found that several cancers, neurological diseases, pulmonary diseases, cardiovascular diseases, and rheumatoid arthritis, among others, are critically associated with CHI3L1 (Fig. 1). Thus, here, we discuss the significant roles of CHI3L1 in the development of cancers (lung, liver, and colon), neurological diseases (Alzheimer’s disease, schizophrenia, etc.), cardiovascular diseases, and rheumatoid arthritis; moreover, we provide some information pertaining to the significance of CHI3L1 in the most common autoimmune disease among Korean children, i.e., atopy.

## Properties of chitinase-like proteins

The most studied mammalian chitinases are chitotriosidase (CHIT1) and acidic mammalian chitinase (AMCase), which are both true chitinases, whereas CHI3L1, chitinase-3-like 2 (CHI3L2), oviductal glycoprotein 1 (OVGP1), and stabilin-1 interacting chitinase like protein (S1-CLP) have the ability to bind chitin but not to degrade it. The properties of CLPs, including CHI3L1, are summarized in Table 1.

**Table 1:** Properties of CLP isoforms.

	CHI3L1	CHI3L2	OVGP1	S1-CLP
Biological activities	Carbohydrate and chitin binding	Carbohydrate and chitin binding	Saccharides and oligosaccharides binding	Oligosaccharide, chitin and protein binding
Expressing cells	Neutrophils, synoviocytes, monocytes/macrophages, osteoclasts, endothelial cell, hepatic stellate cells, smooth muscle cells, and many cancer cells	Synovial fibroblasts, human cartilage chondrocytes, and cancer cells	Oviductal epithelial cell	Macrophages, monocytes, lymphocytes, and sinusoidal endothelial cells, cancer cells
Possible functions	Regulation of cell proliferation, adhesion, migration, and activation Activation of macrophages Regulation of immune-response in inflammation and cancer	Cartilage biogenesis Type II collagen expression Regulation of immune-response	Supportive role in fertilization and embryo development	A ligand for the multifunctional receptor stabilin-1, macrophage inflammatory regulator, pathogen sensing, endotoxin neutralization
Associated diseases	Pneumonia, rheumatoid arthritis, breast cancer, colon cancer, ovarian carcinoma, hepatocellular carcinoma, lung cancer, atherosclerosis, atopy, liver fibrosis, depression, schizophrenia, and Alzheimer’s disease, Parkinson disease, obesity, diabetes, asthma etc.	Rheumatoid arthritis, Osteoarthritis, Alzheimer’s disease, renal, glioma and breast cancer	Ovarian cancers, mucinous carcinomas’ hypertension, endometriosis	Rheumatoid arthritis, osteoarthritis chronic obstructive pulmonary disease, sarcoidosis

**Fig. 2:**
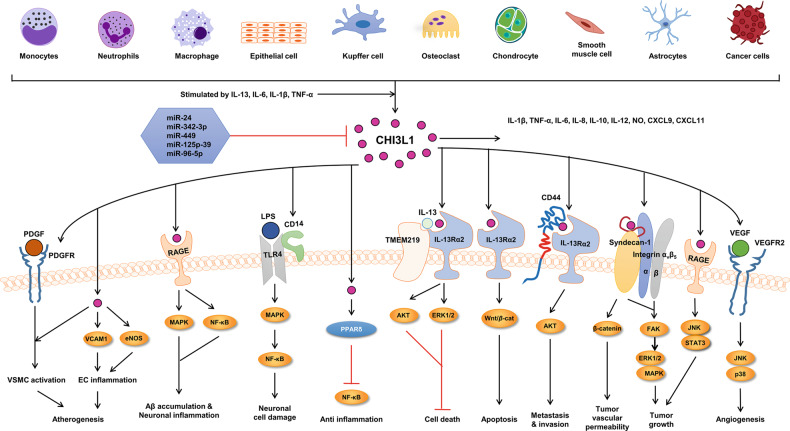
The role of CHI3L1 in signaling pathways for the development of various diseases. Various cells, such as monocytes, neutrophils, macrophages, epithelial cells, Kupffer cells, osteoclasts, chondrocytes, smooth muscle cells, astrocytes, and cancer cells, produce CHI3L1 by the stimulation of several interleukins, such as IL-13, IL-6, IL-1β, and TNF-α. CHI3L1 expression is inhibited by miR-24, miR-342-3p, miR-449, miR125p-39, and miR-96-5p. The activated cells can release (or produce) IL-1β, TNF-α, IL-6, IL-8, IL-10, IL-12, NO, CXCL9, and CXCL11. In the development of atherogenesis, CHI3L1 directly induces PDGF and PDGFR expression to activate VSMCs and/or directly induces VCAM1 and eNOS expression in the cytosol to cause EC inflammation. In the development of neurodegenerative diseases, CHI3L1 activates the MAPK and NF-кB signaling pathways to induce Aβ accumulation and neuronal inflammation via RAGE in either astrocytes or neurons. In neuronal cells, CHI3L1 activity leads to increased expression of CD14 and TLR4 through the MAPK and NF-кB signaling pathways to damage neurons. In cancer development, several receptors and signaling pathways are involved in these processes. CHI3l1 binds to its receptor IL-13Rα2 by associating with TMEM219 directly or by physically interacting with CD44. The TMEM219-dependent pathway prevents cell death by activating the ERK1/2 and AKT pathways, but direct interaction with IL-13Rα2 causes apoptosis through activation of Wnt/β-catenin. Physical interaction with CD44 activates the AKT pathway to induce metastasis and invasion. The membrane receptors syndecan-1 and integrin α_v_β_5_ trigger CHI3L1 signaling pathways, causing tumor vascular permeability and growth by activating the β-catenin, FAK, and ERK 1/2 (MAPK) pathways. CHI3L1 also binds to RAGE and thus activates the FAK and STAT3 pathways, inducing tumor growth. In addition, CHI3L1 elevates the expression of VEGF and its receptor to cause angiogenesis via the activation of the JNK and p38 signaling pathways.

Among them, CHI3L1 has been called YKL-40 in humans and breast regression protein 39 (BRP-39) in mice^10^. CHI3L1 is derived from the three N-terminal amino acids present on the secreted form and its molecular mass. Since its initial detection in the culture supernatant of the MG63 osteosarcoma cell line, it has been subsequently discovered in human chondrocytes, synoviocytes, and vascular smooth muscle cells^11–13^. CHI3L1 protein shows high conservation among species, with homologies of 73.3% in mice, 79.6% in rats, 96.6% in monkeys, and 83.8% in sheep. CHI3L1 is a 40-kDa glycoprotein with heparin, chitin, and collagen-binding properties. It acts as a lectin due to its preserved carbohydrate-binding domain, but its ligands are still unknown^14–17^. CHI3L1 is strongly expressed by macrophages in inflammatory diseases, such as rheumatoid arthritis, asthma, liver cirrhosis, encephalitis, stroke, multiple sclerosis, and glioblastoma^18–27^. It is noteworthy that CHI3L1 has the ability to bind to multiple receptors, such as the receptor for advanced glycation end products (RAGE), syndecan-1/αVβ3, interleukin 13 receptor alpha 2 (IL-13Rα2), and VEGFR2, and this binding leads to the activation of several signals related to inflammasome activation, neuronal inflammation, tumor metastasis and invasion, angiogenesis, apoptosis, carcinogenesis, Aβ accumulation, vascular smooth muscle cell activation, endothelial cell inflammation and atherogenesis (Fig. 2)^28–30^.

## Involvement of CHI3L1 in cancers

### Lung cancer

CHI3L1 is known to play a significant role in the development and progression of lung cancer, promoting tumor cell invasion and metastasis. It is upregulated in lung cancer tissues and associated with a poor prognosis in patients with the disease^31–38^. Studies in mice have shown elevated CHI3L1 expression in non-small cell lung cancer (NSCLC) tissues compared to normal lung tissues, and high CHI3L1 levels are correlated with reduced survival in NSCLC patients^39–41^. Moreover, the inhibition of CHI3L1 expression or activity has been shown to reduce lung tumor growth and size, while its overexpression promotes the growth, migration, and invasion of lung cancer cell lines^42–46^.

CHI3L1 has also been reported to have a proinflammatory function, which is thought to contribute to tumor growth and progression. CHI3L1 activates immune cells (macrophages and neutrophils) and recruits them to the tumor site, where they release proinflammatory cytokines and chemokines, promoting inflammation and supporting cancer cell survival^31,37,38,42–46^. CHI3L1 also directly stimulates cancer cells to produce proinflammatory cytokines (IL-6, IL-8, and TGF-β)^28,46–49^. CHI3L1 can activate TGF-β signaling by binding to its receptor, thus leading to the production of proinflammatory cytokines and the recruitment of immune cells, which can promote tumor growth and metastasis^28^. CHI3L1 recruits and polarizes tumor-associated macrophages (TAMs) toward an M2-like phenotype, suppressing antitumor immune responses and promoting angiogenesis^31,50^. M2 TAMs inhibit cytotoxic T cells (CTLs) and natural killer cell activity, impairing tumor cell elimination. CHI3L1 promotes lung cancer progression by inhibiting CTL activation, increasing T-cell exhaustion, and promoting lung metastasis through the modulation of T-cell costimulatory and immune checkpoint molecules (ICOS, ICOSL, CD28, CTLA-4, and PD-L1)^51^. It also negatively regulates Th1 differentiation and enhances Th2 differentiation via IFN-γ signaling^46^. Additionally, CHI3L1 targets IL-13Rα2, activating the PI3K/Akt pathway to increase lung cancer cell proliferation, migration, and invasion^46,48,49,52^. Overall, the interactions of CHI3L1 contribute to inflammation, tumor growth, and survival in lung cancer.

**Fig. 3:**
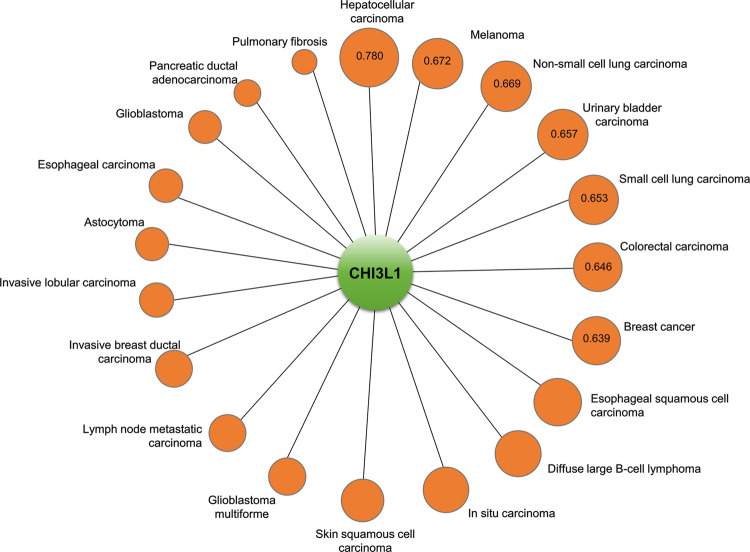
The relationship between CHI3L1 and cancers. The circle sizes are determined based on text-mining scores. The values in the circle symbols are the text-mining scores determined via Open Targets Platform analysis.

**Fig. 4:**
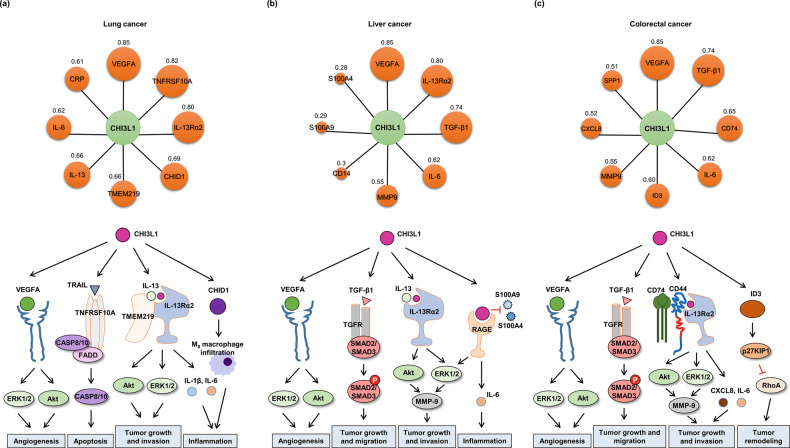
The interaction network between CHI3L1 and its target proteins and the roles of CHI3L1 in signaling pathways for the development of cancers. **a** Lung cancer. Upper panel: The circle sizes are determined based on the score. The values in the circle symbols are the scores determined by STRING analysis. Lower panel: In lung cancer, elevated CHI3L1 levels are linked to increased VEGF expression, promoting angiogenesis and tumor progression. CHI3L1 upregulates VEGF through pathways such as ERK1/2 and Akt. CHI3L1 inhibits apoptosis through interactions with the TRAIL signaling pathway, involving TNFRSF10A and CASP8/CAS10. CHI3L1 can bind to TMEM219 and promote tumor growth and invasion by activating Erk1/2 and Akt signaling. CHI3L1 regulates the IL-13 signaling pathway through IL-13Rα2, leading to increased secretion of cytokines, such as IL-1β and IL-6, which can influence inflammation in lung cancer. There have been no reported associations between CHI3L1 and CHID1. However, it has been observed that CHID1 increases M0 macrophage infiltration. **b** Liver cancer, upper panel; the circle sizes are determined based on the score. The values in the circle symbols are the scores determined by STRING analysis. Lower panel: Elevated CHI3L1 levels increase VEGFA expression through the ERK1/2 and Akt pathways, promoting angiogenesis in liver cancer. CHI3L1 may promote tumor growth and migration by interacting with TGF-β1 and TGFR, thereby activating the SMAD2/SMAD3 signaling pathway. CHI3L1 interacts with IL-13 and IL-13Rα2 to activate the IL-13 signaling pathway, including the AKT and ERK1/2 pathways. Additionally, CHI3L1 affects MMP9, promoting tumor invasion and metastasis. CHI3L1 interacts with RAGE, leading to an increase in IL-6 secretion and inflammation. On the other hand, CHI3L1 may negatively regulate S100A9 and S100A4, which means that its interaction with S100A9 and S100A4 results in a reduction in the proinflammatory effects of S100A9 and S100A4 in liver cancer. **c** Colorectal cancer. Upper panel: The circle sizes are determined based on the score. The values in the circle symbols are the scores determined by STRING analysis. Lower panel: CHI3L1 leads to an increase in VEGF/VEGFA expression through the activation of the ERK1/2 and Akt pathways, promoting angiogenesis and tumor progression in colorectal cancer. CHI3L1 has the potential to enhance tumor growth and migration by interacting with TGF-β1 and TGFR, which subsequently activates the SMAD2/SMAD3 signaling pathway. CHI3L1 interacts with CD44 and IL-13Ra2, activating pathways such as AKT and ERK1/2, which in turn promote tumor growth and invasion. Additionally, CHI3L1 affects MMP-9, contributing to tumor invasion. Furthermore, CHI3L1 induces the secretion of CXCL8 and IL-6, which are proinflammatory cytokines that can also play a role in tumor progression and invasion. The relationship between CHI3L1 and ID3 is not well established. However, the interplay between ID3-induced p27KIP1 and RhoA inhibition can contribute to tumor remodeling in colorectal cancer.

To determine whether CHI3L1 is actually a viable candidate therapeutic approach (as a potential drug target) in lung cancer, we examined the association between CHI3L1 and lung cancer using the Open Targets Platform. The text-mining score of NSCLC and CHI3L1 was 0.669, which indicates a high association (Fig. 3 and Table 2). In addition, the text-mining score of CHI3L1 and small cell lung carcinoma was 0.653 (Table 2). If we sum the text-mining scores listed for NSCLC and small cell lung cancer, lung cancer may be the top disease related to CHI3L1. The text-mining scores of epidermal growth factor receptor (EGFR) and vascular endothelial growth factor (VEGF), which have been widely studied as well-known NSCLC diagnostic marker proteins, were 0.891 and 0.790, respectively. However, the overall association score for the association between CHI3L1 and NSCLC was 0.083, whereas the overall association scores of EGR and VEGF were 0.854 and 0.602, respectively (Table 2). Despite the limited information on its genetic or functional aspects compared with EGFR or VEGFA, CHI3L1 had a relatively high text-mining score. Consequently, further genetic or functional studies of CHI3L1 in NSCLC are needed. Next, we used STRING to identify target proteins associated with CHI3L1. Proteins were classified based on this approach to identify proteins associated with CHI3L1 in lung cancer, which could suggest potential target proteins that are functionally related to CHI3L1. As a result of the STRING analysis, the proteins associated with CHI3L1 in lung cancer were identified in the order of VEGFA, TNF Receptor Superfamily Member 10a (TNFRSF10A), IL-13Rα2, chitinase-domain-containing 1 (CHID), IL-13, Transmembrane Protein 219 (TMEM219), IL-6, and C-reactive protein (CRP) (Fig. 4a upper panel). Next, we investigated the signaling pathways involving CHI3L1 and its target proteins. The signaling pathway between CHI3L1 and its target proteins in NSCLC is illustrated in the lower panel of Fig. 4a. Based on the preliminary findings from the Open Targets Platform and STRING analyses, CHI3L1 exhibits interesting potential as a candidate biomarker for further investigation in lung cancer prediction. Our previous study found that K284-6111 and Ebractenoid F, which are small-molecule inhibitors of CHI3L1 activity, yielded promising results in preclinical studies regarding the inhibition of the growth and metastasis of lung cancer cells^52,53^. Anti-CHI3L1 antibodies specifically target CHI3L1 and prevent it from interacting with its receptors, thereby inhibiting its signaling pathways and lung tumor growth^31^. VEGF and EGFR antibodies and programmed cell death protein 1 inhibitors include bevacizumab, ramucirumab, cetuximab, nivolumab, and pembrolizumab. The standard dosage of these antibodies and inhibitors is 2−15 mg/kg twice a week for 3−4 weeks^54–58^. These antibodies at the indicated doses afford a tumor growth inhibition of approximately 50%. However, our previous study showed that the injection of 0.5 mg/ml of an anti-CHI3L1 antibody twice a week for 4 weeks reduced lung tumor growth by more than 80%. Overall, given the strong evidence linking CHI3L1 to lung cancer formation and progression, targeting this protein may be a promising strategy for the development of new therapies and diagnostic tools for this deadly disease.

**Table 2:** Text mining score and overall association score of cancer types associated with CHI3L1, EGFR, and VEGF on the Open Targets Platform.

CHI3L1-cancer			EGFR-cancer			VEGF-cancer		
Name	Text Mining	Overall Association Score	Name	Text Mining	Overall Association Score	Name	Text Mining	Overall Association Score
Hepatocellular carcinoma	0.780	0.095	Non-small cell lung carcinoma	0.891	0.854	Non-small cell lung carcinoma	0.790	0.602
Melanoma	0.672	0.082	Lung cancer	0.813	0.755	Breast cancer	0.786	0.605
Non-small cell lung carcinoma	0.669	0.083	Glioblastoma multiforme	0.795	0.729	Hepatocellular carcinoma	0.785	0.581
Urinary bladder carcinoma	0.657	0.080	Lung adenocarcinoma	0.788	0.728	Neoplasm	0.784	0.569
Small cell lung carcinoma	0.653	0.079	Head and neck squamous cell carcinoma	0.767	0.691	Glioblastoma multiforme	0.779	0.596
Colorectal carcinoma	0.646	0.079	Neoplasm	0.716	0.622	Renal cell carcinoma	0.750	0.535
Breast cancer	0.639	0.078	Squamous cell carcinoma	0.701	0.607	Multiple myeloma	0.653	0.135
Esophageal squamous cell carcinoma	0.628	0.076	Breast cancer	0.699	0.612	Melanoma	0.641	0.330
Diffuse large B-cell lymphoma	0.608	0.074	Squamous cell lung carcinoma	0.683	0.624	Lung adenocarcinoma	0.594	0.366
In situ carcinoma	0.604	0.073	Breast carcinoma	0.674	0.632	Cancer	0.571	0.150
Skin squamous cell carcinoma	0.565	0.069	Metastatic colorectal cancer	0.638	0.555	Esophageal adenocarcinoma	0.554	0.103
Glioblastoma multiforme	0.494	0.069	Pancreatic carcinoma	0.604	0.625	Osteosarcoma	0.538	0.117
Lymph node metastatic carcinoma	0.490	0.060	Glioma	0.595	0.572	Esophageal squamous cell carcinoma	0.532	0.115
Invasive breast ductal carcinoma	0.489	0.059	Lung carcinoma	0.578	0.564	Glioblastoma	0.526	0.114
Invasive lobular carcinoma	0.456	0.055	Colorectal adenocarcinoma	0.573	0.682	Intrahepatic cholangiocarcinoma	0.525	0.111
Esophageal carcinoma	0.447	0.054	Thyroid carcinoma	0.572	0.455	Acute myeloid leukemia	0.521	0.113
Astrocytoma	0.439	0.055	Brain cancer	0.562	0.437	Nonpapillary renal cell carcinoma	0.520	0.113
Glioblastoma	0.435	0.053	Brain neoplasm	0.556	0.428	Squamous cell carcinoma	0.517	0.131
Pancreatic ductal adenocarcinoma	0.355	0.046	Head and neck malignant neoplasia	0.517	0.646	Adenomyosis	0.513	0.087
Pulmonary fibrosis	0.331	0.040	Colorectal carcinoma	0.515	0.445	Triple-negative breast cancer	0.500	0.133

### Liver cancer

CHI3L1 has also been shown to be involved in the development and progression of liver cancer. In fact, CHI3L1 is expressed at the highest level in the liver among all tissues examined. Previous studies have reported that CHI3L1 is upregulated in patients with liver tumors and that high CHI3L1 expression is associated with a poor prognosis and decreased survival rates^59–63^. The overexpression of CHI3L1 was shown to increase tumor growth, whereas CHI3L1 knockdown inhibited the growth and invasion capacity of hepatocellular carcinoma (HCC) tumors in mice. CHI3L1 was also upregulated in several HCC cells^64^.

CHI3L1 promotes an inflammatory response in liver cancer cells by activating signaling pathways that induce the production of proinflammatory cytokines, such as IL-6 and TNF-α. This can contribute to tumor growth and progression by triggering angiogenesis, cell proliferation, and cell survival. A study explored whether a combination of three inflammatory biomarkers (CHI3L1, IL-6, and CRP) along with carcinoembryonic antigen and carbohydrate antigen 19-9 could predict the prognosis in patients with liver metastases after resection^62^. Moreover, another study found that CHI3L1 increased the TNF-α-induced proliferation, migration, and invasion of liver cancer cells^65^. Furthermore, CHI3L1 regulates signaling pathways in liver cancer that are involved in tumor growth and progression. In fact, CHI3L1 can modulate TGF-β signaling in liver cancer through the regulation of SMAD Family Member-2 (SMAD-2) and SMAD-3^64^. Therefore, targeting CHI3L1 may inhibit liver tumor growth and metastasis.

As stated above, targeting CHI3L1 may have therapeutic potential in liver cancer. We used the Open Targets Platform to validate the link between CHI3L1 and liver cancer. The text-mining scores of CHI3L1, EGFR, and VEGF in hepatocellular carcinoma were 0.780, 0.393, and 0.785, respectively, indicating a higher association of CHI3L1 and VEGF in the disease than for EGFR (Table 2). The overall association scores of CHI3L1, EGFR, and VEGF were 0.095, 0.389 and 0.581, respectively (Table 2). CHI3L1 had lower overall association scores than EGFR and VEGF because there is less genetic and functional evidence for CHI3L1. The high text-mining score of CHI3L1 suggests a significant association with liver cancer, similar to those of the representative marker proteins EGFR and VEGF. Subsequently, a target protein connected to CHI3L1 in liver cancer was discovered using STRING analysis, which revealed that the following proteins were linked to CHI3L1 in liver cancer: VEGFA, IL-13Rα2, TGF-β1, matrix metalloproteinase (MMP)-9, IL-6, cluster of differentiation 14 (CD14), S100 Calcium Binding Protein A6 (S100A6), and S100A4 (Fig. 4b upper panel). The lower panel of Fig. 4b illustrates the liver cancer signaling pathway between CHI3L1 and its target proteins. Overall, the association between CHI3L1 and these target proteins in liver cancer suggests that CHI3L1 plays an important role in liver cancer progression and metastasis and that targeting CHI3L1 is a potential therapeutic approach for liver cancer.

### Colorectal cancer

CHI3L1 is upregulated in colon tumor tissues and has been proposed as a potential biomarker of colorectal cancer. CHI3L1 is significantly upregulated in patients with colorectal cancer compared with healthy individuals^66,67^. Moreover, overexpressing CHI3L1 in colorectal cancer cells was shown to enhance colon tumor growth in mice^68^. Furthermore, CHI3L1 overexpression significantly enhanced the proliferation of SW480 cells. Conversely, the knockdown of CHI3L1 by RNA interference or neutralization by an anti-CHI3L1 antibody strongly suppressed the CHI3L1-induced migration and tube formation of human colorectal cancer cells^68^.

CHI3L1 also regulates the expression of proinflammatory cytokines in colorectal cancer. Previous studies have shown that CHI3L1 can stimulate the migration and invasion of cancer cells by modulating the tumor microenvironment^68,69^. CHI3L1 treatment significantly increases the secretion of IL-8, monocyte chemoattractant protein-1, and VEGFA from cancer-associated fibroblasts^69^. Another study showed that the upregulation of these cytokines and angiogenic factors by CHI3L1 was mediated by the activation of the MAPK signaling pathways^68^. In addition, secreted CHI3L1 activates the NF-кB signaling pathways in cancer cells and macrophages, thus leading to a protumor tumor microenvironment characterized by activated M2 macrophages^50^. In addition, CHI3L1 overexpression leads to a reduction in S100A9 expression, increases cell sensitivity to cetuximab and promotes cell proliferation by downregulating p53 and upregulating EGFR^70^.

Colorectal cancer is the fourth most common cancer in Korea, after thyroid, lung, and stomach cancer. In addition, multiple studies have found that Koreans have the highest rate of colorectal cancer in the world. We investigated the relationship between CHI3L1 and colorectal cancer using the Open Targets Platform. The text-mining scores of CHI3L1, EGFR, and VEGF were 0.646, 0.638, and 0.307, respectively (Table 2). The overall association scores of CHI3L1, EGFR and VEGF with colorectal cancer were 0.079, 0.555, and 0.596, respectively. While drugs targeting EGFR and VEGFA have been extensively studied for colorectal cancer, there are currently no reported drugs for this disease targeting CHI3L1. Therefore, further research on the development of drugs targeting CHI3L1 in colorectal cancer is necessary. Next, we analyzed the target proteins of CHI3L1 in colorectal cancer. We identified several potential CHI3L1 target proteins, including VEGFA, TGF-β1, Cluster of Differentiation 74 (CD74), IL-6, inhibitor of DNA binding 3 (ID3), MMP9, CXCL8, and SPP1, in this type of cancer (Fig. 4c upper panel). The signaling pathway between CHI3L1 and its target proteins in colorectal cancer is depicted in the lower panel of Fig. 4c. The putative target proteins of CHI3L1 in colorectal cancer are involved in diverse biological processes related to the development and progression of the disease.

## Involvement of CHI3L1 in neurological diseases

The expression of CHI3L1 is increased in patients with various neurological diseases, including Alzheimer’s disease (AD), amyotrophic lateral sclerosis, multiple sclerosis, and schizophrenia (SCZ)^71^. According to the comprehensive Open Targets Platform and text-mining scores, AD is the neurological disease most strongly associated with the *CHI3L1* gene. In addition, ALS and SCZ are also associated with the *CHI3L1* gene in the neurological disease category. CHI3L1 is widely expressed in all regions of the human brain and is particularly found in activated microglia and astrocytes, making it a potential biomarker for neurological diseases^72^. The intricate interactions of the immune system with the brain hold significant therapeutic potential in neurological diseases, and CHI3L1 has emerged as a promising therapeutic target in this context. This section describes the associations and related target genes of CHI3L1 in neurological diseases.

### Alzheimer’s disease

CHI3L1 is a putative marker of neuroinflammation and has potential prognostic utility as a preclinical biomarker of AD^73^. The increased plasma concentration of CHI3L1 in patients with early AD suggests its usefulness as an early diagnostic indicator^74–77^. The upregulation of CHI3L1 is linked to the immune activation of microglia and neuronal death, and it is also responsive to hippocampal neuron injury^78,79^. In addition, increased expression of CHI3L1 in both healthy women and in brain regions affected by late-onset Alzheimer’s disease indicates a possible explanation for the higher prevalence of AD in women than in men^80–83^. Recent research has shown that CHI3L1 plays a role in the formation of amyloid plaques, which are a hallmark of AD pathology^72^. CHI3L1 is primarily expressed in astrocytes in the brains of AD patients and is linked to neuroinflammation in the white matter and cognitive decline^84,85^.

CHI3L1 is upregulated in the hippocampus of 9-month-old 5xFAD mice, which are AD model animals expressing human amyloid beta precursor protein^86^ and presenilin 1 (*PSEN1*) transgenes^87^. The activation of NF-κB has been observed in the brains of AD patients, and disrupting the NF-κB pathway decreases β-site APP-cleaving enzyme 1 expression and Aβ generation, leading to memory deficits and reduced neurogenesis in vivo^88–90^. Therefore, targeting CHI3L1 is a potential therapy for AD by reducing neuroinflammation. Furthermore, animal studies have shown that the knockout of CHI3L1 proteins can significantly decrease AD pathogenesis, suppress glial phagocytic activation, and reduce amyloid accumulation^72^.

Several candidate genes and signals associated with CHI3L1 and AD have been proposed. Increased levels of CHI3L1, APOE, and visinin-like 1 (VSNL1) in the cerebrospinal fluid (CSF) have been associated with AD^91,92^. IL-1β and IL-6, which can upregulate CHI3L1 in astrocytes through STAT3-dependent signaling, have also been implicated in neuroinflammatory dementias^93^. Evidence indicates a shared neuroinflammatory profile among neurodegenerative dementias, where levels of CHI3L1, CHIT1, and GFAP are significantly increased in AD^94^. These findings suggest that several genes are associated with CHI3L1-related neuroinflammation and AD. The inhibition of CHI3L1 using K284-6111 has been reported to block this process, prevent neuroinflammation, and attenuate cognitive impairments^95,96^.

**Fig. 5:**
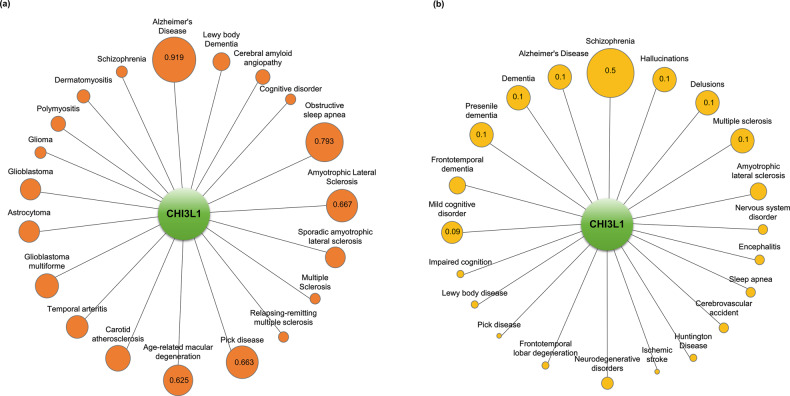
The relationship between CHI3L1 and neurological diseases. **a** The circle sizes are determined based on the text-mining score. The values in circle symbols are the text-mining scores determined via Open Targets Platform analysis. **b** The circle sizes are determined based on the gda score. The values in the circle symbols are the gda scores determined via DisGeNET analysis.

**Fig. 6:**
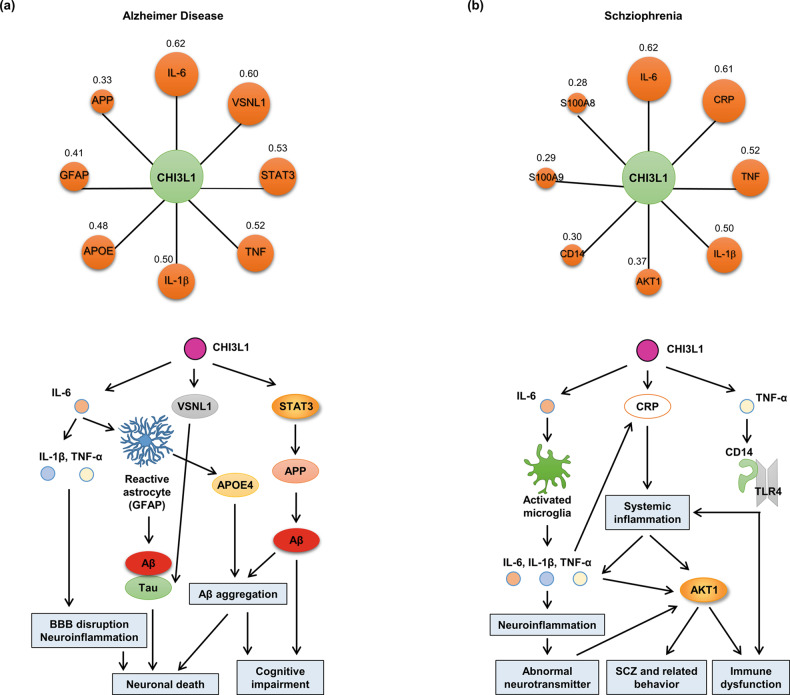
The interaction network between CHI3L1 and its target proteins and the roles of CHI3L1 in signaling pathways for the development of neurological diseases. **a** Alzheimer’s disease. Upper panel: The circle sizes are determined based on the score. The values in the circle symbols are the scores determined by STRING analysis. Lower panel: CHI3L1 regulates IL-6, inducing an increase in IL-1β and TNF-α. This pathway leads to the disruption of the blood‒brain barrier (BBB) and triggers neuroinflammation, ultimately resulting in neuronal death. IL-6 activates astrocytes, and reactive astrocytes (with increased GFAP) induce Aβ aggregation and Tau phosphorylation. They can also induce Aβ aggregation through the ApoE4 pathway, ultimately leading to neuronal death and cognitive impairment. CHI3L1 downregulates VSNL1 and increases Tau phosphorylation. CHI3L1 activates STAT3 and increases APP expression in neuronal cells, resulting in Aβ aggregation and cognitive impairment. **b** Schizophrenia. Upper panel: The circle sizes are determined based on the score. The values in the circle symbols are the scores determined by STRING analysis. Lower panel: CHI3L1 regulates IL-6, inducing microglial activation. Activated microglia express IL-6, IL-1β, and TNF-α, which leads to neuroinflammation and triggers abnormal neurotransmitter signaling. Ultimately, this process results in schizophrenia and related behavioral and immune dysfunction. The association score between CHI3L1 and CRP is high, indicating a strong correlation. However, the specific interaction between these two factors has not yet been identified. CRP is increased by inflammatory cytokines (IL-6, IL-1β, and TNF-α) and induces systemic inflammation, leading to immune dysfunction via the AKT1 pathway. CHI3L1 regulates TNF-α and induces immune dysfunction through the CD14-TLR4 pathway.

We also investigated the relationship between CHI3L1 and AD using the Open Targets Platform. The text-mining score of CHI3L1 (0.919) was the highest score, and the overall association score (0.133) was the second highest score among the neurological diseases (Fig. 5a and Table 3). These data suggest that CHI3L1 is a significant target for AD. The text-mining and overall association scores of other AD targets, such as APP (0.995; 0.824), were consistent (Table 3). These data indicate the necessity for the study of genetic function and drug development for CHI3L1. Next, we analyzed the target proteins of CHI3L1 in AD using the STRING system. We identified several potential CHI3L1 target proteins, including APP, PSEN1, APOE, IL-1β, VSNL1, TNF, IL-6, and GFAP, in AD (Fig. 6a upper panel). The lower panel of Fig. 6a depicts the signaling pathway between CHI3L1 and its target proteins in AD. These putative target proteins of CHI3L1 in AD are involved in diverse biological processes that contribute to neurodegenerative processes.

**Table 3:** Text mining score and overall association score of neurological diseases associated with CHI3L1, APP, and DRD2 on the Open Targets Platform.

CHI3L1-neurological diseases			APP-neurological diseases				DRD2-neurological diseases		
Name	Text Mining	Overall Association Score	Name	Text Mining	Overall Association Score	Name		Text Mining	Overall Association Score
Alzheimer disease	0.919	0.113	Alzheimer disease	0.995	0.824		Schizophrenia	0.942	0.732
Obstructive sleep apnea	0.793	0.096	Neuroblastoma	0.931	0.113		Parkinson disease	0.826	0.636
Amyotrophic lateral sclerosis	0.667	0.081	Dravet syndrome	0.898	0.109		Alcohol dependence	0.779	0.611
Pick disease	0.663	0.081	Parkinson disease	0.774	0.094		Psychosis	0.757	0.624
Age-related macular degeneration	0.625	0.076	Experimental autoimmune encephalomyelitis	0.694	0.084		Alzheimer disease	0.668	0.428
Carotid atherosclerosis	0.525	0.064	Schizophrenia	0.693	0.092		Insomnia	0.648	0.097
Glioblastoma multiforme	0.493	0.069	Autism	0.690	0.083		Autism	0.631	0.624
Temporal arteritis	0.464	0.056	Cerebral amyloid angiopathy	0.689	0.361		Prolactin-producing pituitary gland adenoma	0.613	0.083
Astrocytoma	0.438	0.055	Brain ischemia	0.607	0.073		Glioblastoma	0.608	0.073
Glioblastoma	0.435	0.053	Glioblastoma	0.513	0.062		Attention deficit hyperactivity disorder	0.572	0.658
Sporadic amyotrophic lateral sclerosis	0.425	0.052	Familial Alzheimer disease	0.455	0.055		Experimental autoimmune encephalomyelitis	0.488	0.059
Lewy body dementia	0.365	0.044	Huntington disease	0.419	0.051		Lewy body dementia	0.425	0.051
Polymyositis	0.312	0.038	Nervous system disease	0.331	0.040		Conduct disorder	0.419	0.556
Cerebral amyloid angiopathy	0.305	0.037	Stroke	0.317	0.392		Huntington disease	0.417	0.393
Dermatomyositis	0.277	0.034	Age-related macular degeneration	0.291	0.035		Post-traumatic stress disorder	0.384	0.585
Glioma	0.238	0.029	Amyotrophic lateral sclerosis	0.289	0.050		Catalepsy	0.377	0.045
Schizophrenia	0.237	0.377	Fragile X syndrome	0.268	0.032		Dyslexia	0.364	0.044
Multiple sclerosis	0.221	0.027	Neurodegenerative disease	0.264	0.032		Cocaine dependence	0.330	0.469
Cognitive disorder	0.218	0.027	Tauopathy	0.228	0.027		Fabry disease	0.305	0.0371
Relapsing-remitting multiple sclerosis	0.213	0.026	Brain injury	0.226	0.027		Migraine disorder	0.282	0.590

### Schizophrenia

Schizophrenia, which affects approximately 1% of the population, is a prevalent mental disorder. Schizophrenia results from a combination of genetic, environmental, and neurobiological factors; however, its specific mechanism and function remain unclear. On multiple discovery platforms (such as DisGeNET and the Open Targets Platform), SCZ has been found to be most strongly associated with CHI3L1. For example, CHI3L1 was shown to be upregulated in the hippocampus of patients with SCZ^97^. Since then, clinical studies have shown that single-nucleotide polymorphisms (SNPs) of the *CHI3L1* gene are associated with the risk of SCZ^98,99^. Specifically, the G allele of a SNP in the *CHI3L1* gene has been found to be linked to a higher expression of the CHI3L1 protein in the serum of patients with SCZ^100^. However, this genetic association between the *CHI3L1* gene and SCZ has failed to be confirmed in Japanese and Chinese populations^101^. Therefore, there is a lack of in vivo and in vitro studies to demonstrate the relationship between CHI3L1 and SCZ.

Several studies have reported that CHI3L1 is involved in inflammatory and immune responses, including the regulation of inflammatory cytokines and the proliferation and activation of immune cells^102,103^. The importance of inflammation in the risk of developing SCZ has been increasingly recognized^104–106^. The AKT1–GSK3β pathway, which is related to oxidative stress and inflammation, has been suggested to be relevant to the expression of CHI3L1 in SCZ^107^. CHI3L1 induces the production of inflammatory cytokines, whereas AKT signaling is activated by PI3K through SCZ-associated cytokine receptors, colony stimulating factor 2 receptor subunit alpha, and IL-13Rα2^108,109^. The overexpression of CHI3L1, together with other proteins (SERPINA3, IFITM 1, IFITM2, and CD14), has been associated with prefrontal dysfunction in SCZ^110,111^. In first-episode psychosis, increased plasma levels of MCP-1 and CHI3L1 were suggestive of immune reactions in SCZ^112^. We also recently reported that the transmembrane inflammatory cytokine TNF-α may be significant for the development of SCZ^113^. A recent study indicated that CHI3L1 is associated with subsequent type 2 diabetes and low-grade chronic inflammation in patients with SCZ. We next investigated the relationship between CHI3L1 and SCZ using the Open Targets Platform. The text-mining score of CHI3L1 was 0.237 (Fig. 5a and Table 3). Moreover, the overall association score (0.377) was the highest score among neurological diseases. The DRD2 target may be the most promising target for the development of drugs for SCZ because it had the highest text-mining and overall association scores (0.942; 0.732) (Table 3). APP had a low overall association score (0.092), even though it showed a relatively high text-mining score (0.693), indicating that it has lower therapeutic potential as a target for SCZ than DRD2. Although CHI3L1 has a lower text mining score than APP, its overall association score is relatively high, indicating a potential for further research. In addition, we found that CHI3L1 was the most interesting target regarding the development of drugs for SCZ because, according to the DisGeNET analysis^114^, the gene–disease association (gda) score of CHI3L1 was the highest for its relationship with SCZ (Fig. 5b). These data suggest that CHI3L1 is a potential target in SCZ. Next, we analyzed the target proteins of CHI3L1 in SCZ. As mentioned previously in this section, we identified several potential CHI3L1 target proteins, including AKT1, CD14, CRP, TNF, IL-1β, IL-6, S100A8, and S100A9, in SCZ (Fig. 6b upper panel). We showed the detailed signaling pathways between the target proteins and CHI3L1 (Fig. 6b lower panel).

### Other neurological diseases

In addition, CHI3L1 has been associated with various other neurological diseases, i.e., Parkinson’s disease (PD), amyotrophic lateral sclerosis, Huntington’s disease, MS, progressive supranuclear palsy, and epilepsy. CHI3L1 has been implicated in neuroinflammation and dopaminergic neuron degeneration in PD^115^. Neuroinflammation was shown to predict nonmotor symptoms and cognitive decline in early PD stages^116^. CHI3L1 expression was shown to be increased in the brain tissues and CSF of LPS-induced PD rats along with inflammatory cytokine release^117^. In amyotrophic lateral sclerosis, CHI3L1 has been shown to promote astrocyte activation and motor neuron death^118^. In Huntington’s disease, CHI3L1 has been suggested as a potential biomarker of disease progression^119^. In patients with MS, CHI3L1 is a promising biomarker of disability because of its elevated levels in the CSF^120^. Moreover, the CSF levels of CHI3L1 are strongly correlated with MS pathology and can distinguish between primary progressive MS and relapsing–remitting MS^121^. Elevated levels of CHI3L1 in the CSF were found to be associated with faster disease progression^122^. In turn, in progressive supranuclear palsy, CHI3L1 was found to be elevated in the CSF and brain tissues and was correlated with disease severity^123^. Finally, in epilepsy, CHI3L1 has been suggested to be a potential marker of seizure severity and epilepsy type^124^. Further studies are necessary to clarify the mechanisms by which CHI3L1 contributes to the pathogenesis of these diseases and its potential as a biomarker and therapeutic target.

## Involvement of CHI3L1 in cardiovascular diseases

Cardiovascular disease (CVD) is a collective term for a group of disorders of the heart and blood vessels. These diseases are the largest cause of morbidity and premature death worldwide. Specifically, atherosclerosis is a chronic disorder that occurs in the arterial walls and is associated with endothelial dysfunction, foam cell formation, cholesterol deposition, inflammation, ECM synthesis, smooth muscle cell biological transition, and immature neovascularization of plaques in disease initiation and development^125–128^. In a previous histopathological study that was performed over 20 years ago, strongly upregulated expression of CHI3L1 was detected in distinct subtypes of macrophages in advanced atherosclerotic lesions via in situ hybridization^129^. Subsequently, several clinical studies have reported that an elevated serum CHI3L1 level compared with that of healthy controls is associated with an increased risk of hypertension, endothelial dysfunction, vascular injury, angiographic lesion progression, carotid atherosclerosis, peripheral artery disease, atherosclerosis, large-artery atherosclerotic stroke, coronary artery disease, and cardiovascular complications in patients with diabetes, heart or kidney transplant recipients, and patients with sleep apnea syndrome^86,130–151^, suggesting CHI3L1 as a diagnostic marker for CVD. However, the pathophysiological importance of CHI3L1 for CVD, especially hypertension and atherosclerosis, and the correlation between CHI3L1 and its associated genes have not been sufficiently discussed. This section describes the present knowledge about the role of CHI3L1 in CVD and discusses its relationship with target genes.

### Hypertension

CHI3L1 was reported to be associated with the risk of hypertension. Several studies have demonstrated that CHI3L1 is associated with the severity of hypertension, insulin-resistant hypertension, portal hypertension, and several heart disease-related hypertension^152–157^. A population-based nested case‒control study, a cohort study of 700 prehypertensive Chinese subjects and a 1:1 matched prospective cohort study with 507 Chinese subjects showed that CHI3L1 could be a biomarker for predicting the risk of hypertension^158^. A single-center prospective observational cohort study with 327 hypertensive patients showed elevated serum CHI3L1 levels^159^. Elevated circulating CHI3L1 levels are associated with hypertension in obstructive sleep apnea patients and cirrhotic portal hypertension (CPH), indicating the potential of CHI3L1 as a specific biomarker for hypertension^155,160^. CHI3L1 may serve as a diagnostic biomarker for systemic sclerosis with pulmonary arterial hypertension and pulmonary hypertension associated with bronchopulmonary dysplasia^152,156,161–165^.

Although several recent clinical studies have shown that the range of CHI3L1 concentrations in healthy adults is approximately 30–60 ng/mL^130,133,134,143,148,149^, to date, there is no precisely established normal range for the circulating levels of CHI3L1 in healthy individuals. With 121 chronic heart failure (CHF) patients, including hypertensive heart disease patients and 19 age-matched healthy controls, a study showed that serum CHI3L1 levels were significantly elevated in patients with hypertensive heart disease (205 ± 15 ng/mL) compared with healthy controls (163 ± 77 ng/mL)^166^. Ma et al. demonstrated that the serum CHI3L1 results of each group were as follows: nonmicroalbuminuric group, 61.63 ± 18.58 ng/ml; microalbuminuric group, 98.78 ± 19.83 ng/ml; and healthy controls, 37.85 ± 14.12 ng/ml^167^. In a study with 60 essential hypertension patients and 30 healthy subjects, serum CHI3L1 levels were significantly higher in the essential hypertension group than in the control group [51.7 (35.6–341.9) µg/L vs. 33.2 (23.3–167.3) µg/L]. These levels were also significantly higher in essential hypertension patients with metabolic syndrome than in essential hypertension patients without metabolic syndrome (152.3 µg/L vs. 94.2 µg/L). Thus, serum CHI3L1 levels might be used as a biomarker reflecting inflammation status in hypertensive patients.

Recent evidence has implicated CHI3L1 in patients with inflammatory diseases and cardiometabolic disorders, making it potentially useful to evaluate disease severity, prognosis and survival^152,168–171^. Using 40 age- and sex-matched dipper hypertensive patients and 40 nondipper hypertensive patients, it was found that nondippers had significantly increased epicardial adipose tissue (EAT) thickness and higher CHI3L1 and high-sensitivity CRP levels than dippers^172^. A study showed that bitransgenic mice that overexpressed human heme oxygenase-1, an anti-inflammatory gene, showed inhibited macrophage accumulation and activation, an induction of macrophage IL-10 expression, and the prevention of the development of hypoxia-induced pulmonary hypertension^173^. In a spontaneously hypertensive rat (SHR) animal model, it was identified that the gene expression levels of an inflammatory marker, CHI3L1, were higher in SHRs than in normotensive Wistar–Kyoto rats^174^. These studies suggest that neuroinflammation could be significantly associated with hypertension.

**Fig. 7:**
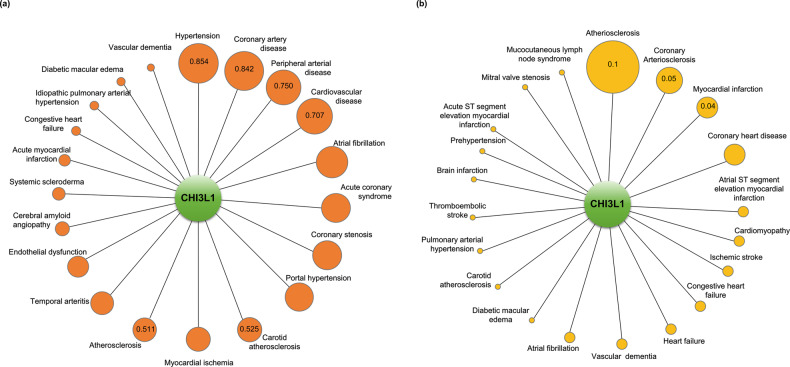
The relationship between CHI3L1 and cardiovascular diseases. **a** The circle sizes are determined based on the text-mining score. The values in the circle symbols are the text-mining scores determined via Open Targets Platform analysis. **b** The circle sizes are determined based on the gda score. The values in the circle symbols are the gda scores determined via DisGeNET analysis.

**Fig. 8:**
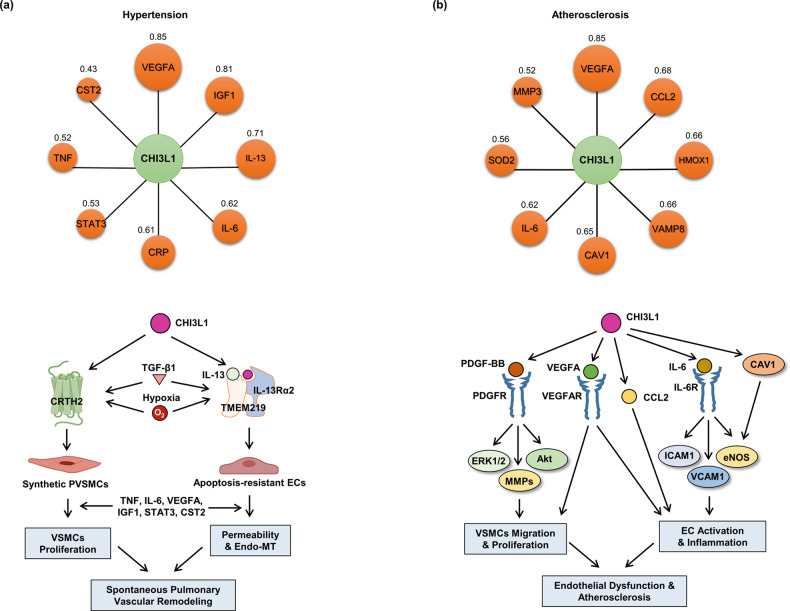
The interaction network between CHI3L1 and its target proteins and the roles of CHI3L1 in signaling pathways for the development of cardiovascular diseases. **a** Hypertension. Upper panel: The circle sizes are determined based on the score. The values in the circle symbols are the scores determined by STRING analysis. Lower panel**:** In the development of hypertension, CHI3L1 stimulates pulmonary artery vascular smooth muscle proliferation through interaction with CRTH2, a G protein-coupled receptor. CHI3L1 binds with IL-13Rα2/TMEM219 and mediates anti-apoptotic effects in pulmonary arterial endothelial cell death through synergy with TGF-ß1 and hypoxia, promoting endothelial permeability and endo-MT transition. This causes spontaneous pulmonary vascular remodeling and hypertension. **b** Atherosclerosis. Upper panel: The circle sizes are determined based on the score. The values in the circle symbols are the scores determined by STRING analysis. Lower panel**:** In atherogenesis, CHI3L1 induces endothelial activation and inflammation through synergy with IL-6 and increases VEGFA and CCL2 levels. CHI3L1 potentiates PDGF-BB-induced vascular smooth muscle cell migration and proliferation and probably elevates the VEGFA signaling pathway. As a result, endothelial dysfunction and the onset of atherosclerosis are induced.

To determine whether CHI3L1 is actually a viable candidate therapeutic strategy as a potential drug target in CVD, we verified the association between CHI3L1 and CVD using the Open Targets Platform. As shown in Table 4, the text-mining score of CHI3L1 in hypertension was 0.854, which represented the highest association among the CVDs (Fig. 7a and Table 4). In turn, the text-mining scores of VCAM1 and ITGAX, which are widely studied and well-known genes in hypertension, were 0.706 and 0.149, respectively (Table 4). The overall association scores (0.104) were also highest among the CADs. In addition, the overall association scores with VCAM1 and ITGAX were also high (0.086 and 0.030). However, DisGeNET analysis showed that CHI3L1 was not closely related (Fig. 7b). Further studies regarding the drug target of CHI3L1 in hypertension are needed. STING analysis revealed that the following proteins were related to CHI3L1 in hypertension: VEGFA, IGF1, IL-13, IL-6, CRP, STAT3, TNF and CST2 (Fig. 8a upper panel). A detailed signaling pathway related to this is shown in Fig. 8a (lower panel).

**Table 4:** Text mining score and overall association score of cardiovascular diseases associated with CHI3L1, VCAM1, and ITGAX on the Open Targets Platform.

CHI3L1-cardiovascular diseases			VCAM1-cardiovascular diseases			ITGAX-cardiovascular diseases		
Name	Text Mining	Overall Association Score	Name	Text Mining	Overall Association Score	Name	Text Mining	Overall Association Score
Hypertension	0.854	0.104	Atherosclerosis	0.876	0.106	Atherosclerosis	0.798	0.097
Coronary artery disease	0.842	0.102	Cardiovascular disease	0.851	0.104	Behcet’s syndrome	0.236	0.029
Peripheral arterial disease	0.750	0.091	Endothelial dysfunction	0.799	0.097	Hypertension	0.149	0.030
Cardiovascular disease	0.707	0.086	Hypertension	0.706	0.086	Coronary artery disease	0.148	0.018
Atrial fibrillation	0.678	0.082	Dilated cardiomyopathy	0.627	0.076	Atrial fibrillation	0.146	0.018
Acute coronary syndrome	0.627	0.076	Duchenne muscular dystrophy	0.602	0.075	Mucocutaneous lymph node syndrome	0.122	0.015
Coronary stenosis	0.626	0.076	Arteriosclerosis	0.538	0.065	Atrial heart septal defect	0.076	0.009
Portal hypertension	0.608	0.074	Stroke	0.533	0.065	Vasculitis	0.065	0.008
Carotid atherosclerosis	0.525	0.064	Coronary artery disease	0.512	0.062	Myocarditis	0.064	0.008
Myocardial Ischemia	0.522	0.063	Vasculitis	0.341	0.042	Carotid atherosclerosis	0.061	0.007
Atherosclerosis	0.511	0.062	Heart failure	0.321	0.039	Abdominal Aortic Aneurysm	0.061	0.007
Temporal arteritis	0.464	0.056	Mucocutaneous lymph node syndrome	0.292	0.035	Congestive heart failure	0.061	0.007
Endothelial dysfunction	0.456	0.055	Systemic scleroderma	0.289	0.035	Arteritis	0.055	0.007
Cerebral amyloid angiopathy	0.305	0.037	Acute coronary syndrome	0.245	0.030	Cardiovascular disease	0.053	0.024
Systemic scleroderma	0.276	0.034	Rheumatic heart disease	0.230	0.028	Systemic scleroderma	0.041	0.005
Acute myocardial infarction	0.249	0.030	Atrial fibrillation	0.229	0.028	Thromboangiitis obliterans	0.036	0.004
Congestive heart failure	0.193	0.023	Pulmonary arterial hypertension	0.212	0.026	Aortic aneurysm	0.035	0.004
Idiopathic pulmonary arterial hypertension	0.188	0.023	Myocardial Ischemia	0.211	0.026	Acute myocardial infarction	0.031	0.004
Diabetic macular edema	0.182	0.022	Acute myocardial infarction	0.197	0.024	Thrombotic disease	0.030	0.004
Vascular dementia	0.155	0.019	Myocardial infarction	0.193	0.023	Coronary atherosclerosis	0.030	0.004

### Atherosclerotic vascular diseases

Serum CHI3L1 levels have been found to be elevated in atherosclerotic vascular diseases. In a study that included 89 patients with symptomatic or asymptomatic carotid atherosclerosis and 20 age-matched healthy controls, the serum CHI3L1 levels were significantly elevated in the patients with carotid atherosclerosis, particularly in symptomatic patients (114.9 ± 10.5 ng/mL), compared with the healthy controls (49.1 ± 3.2 ng/mL)^137^. Interestingly, a recent large-scale study of 302 patients with asymptomatic carotid atherosclerosis based on blood chemistry analysis combined with noninvasive cervical color Doppler ultrasound imaging revealed that high CHI3L1 levels (237.08 ± 67.24 ng/mL) were an independent risk factor for unstable plaque formation^138^. Furthermore, the serum CHI3L1 levels were significantly higher in patients with large-artery atherosclerosis (LLA) stroke than in healthy controls, suggesting that CHI3L1 is an independent prognostic biomarker for the prediction of the clinical outcomes of LLA stroke^146^. One study even suggested that the serum level of CHI3L1 may be a useful initial screening biomarker or subsequent risk indicator for atherosclerosis in children and adolescents^144^. These studies demonstrated that CHI3L1 is closely related to both the early and late phases of atherosclerotic vascular diseases.

A significant positive correlation was reported between CHI3L1 levels and the progression or severity of coronary artery disease. The serum CHI3L1 levels were markedly higher in patients with angiographic lesion progression (123.93 ± 74.01 ng/mL) compared with those without it (71.05 ± 55.14 ng/mL) and were significantly correlated with a change in lumen diameter stenosis and the cumulative coronary obstruction score, suggesting that an increased serum level of CHI3L1 is independently associated with lesion progression in patients with CAD^136^. Even higher levels of CHI3L1 have been documented in patients suffering from peripheral artery disease (PAD). A study involving 612 health-screened subjects found elevated circulating CHI3L1 levels in 86 subjects (406.7 ± 286.6 ng/mL) with PAD, and this finding was positively correlated with inflammatory biomarkers, suggesting that the level of circulating CHI3L1 is significantly associated with the risk of peripheral artery disease^139^. In addition, a recent study that included 365 PAD patients reported that baseline serum CHI3L1 levels were significantly associated with long-term cardiovascular mortality and all-cause mortality^140^.

Atherosclerotic vascular disease is a chronic inflammatory disorder that affects the blood vessel walls, eventually leading to complete obstruction of the blood flow, myocardial infarction, ischemic stroke, and PAD^175,176^. The vascular endothelium is composed of a monolayer of endothelial cells (ECs) that line the lumen of the blood vessels and has emerged as a key regulator of vascular physiological roles, including tight junction function, vascular tone regulation, and cell adhesion and secretion^177,178^. The initiation of atherogenesis is accompanied by EC dysfunction, including vascular inflammation and infiltration, and the stimulating phenotypic change in vascular smooth muscle cells (VSMCs) that is involved in the early pathogenesis of the disease^179,180^. The involvement of CHI3L1 in inflammatory conditions and vascular processes implies that CHI3L1 plays an atherogenic role in endothelial dysfunction^181,182^. A recent study demonstrated that CHI3L1 induces endothelial inflammation and enhances platelet-derived growth factor (PDGF)-induced VSMC migration and proliferation^94^. Another study reported that CHI3L1 ameliorates LPS-induced atherosclerotic responses via PPARδ-mediated suppression of inflammation and endoplasmic reticulum stress and apoptosis, as studied using HUVECs and THP-1 cells^183^. Although the role of CHI3L1 in the pathogenesis of atherosclerotic vascular disease remains understudied, recent research findings suggest that targeting CHI3L1 may provide a novel therapeutic strategy for atherosclerosis.

As shown in Table 4, the text-mining scores of CHI3L1 in coronary artery disease, peripheral arterial disease, carotid atherosclerosis, and atherosclerosis were 0.842, 0.750, 0.525, and 0.511, respectively (Fig. 7a and Table 4). The text-mining scores of VCAM1, which is a widely studied and well-known gene in CVD, in atherosclerosis, cardiovascular disease, endothelial dysfunction, dilated cardiomyopathy, were 0.876, 0.851, 0.799, and 0.627, respectively. The text-mining scores of ITGAX, another well-known gene in CVD, atherosclerosis, Behcet’s syndrome, coronary artery disease, and atrial fibrillation, were 0.798, 0.236, 0.148, and 0.146, respectively (Table 4). The overall association scores of CHI3L1 in coronary artery disease, peripheral arterial disease, carotid atherosclerosis, and atherosclerosis were 0.102, 0.091, 0.064, and 0.062, respectively, which are similar to the overall association scores of VCAM1 and ITGAX (Table 4). Moreover, DisGeNET analysis showed that arteriosclerosis had the highest association with CHI3L1 among CVDs, with a gda score of 0.1 (Fig. 7b). These data indicate that CHI3L1 is highly associated with atherosclerotic vascular diseases. STING analysis revealed that the following proteins were related to CHI3L1 in CVD: VEGFA, CCL2, HMOX1, Vesicle Associated Membrane Protein 8 (VAMP8), Caveolin 1 (CAV1), IL-6, Superoxide dismutase 2 (SOD2), and MMP-3 (Fig. 8b upper panel). The lower panel of Fig. 8b depicts the signaling pathway connecting CHI3L1 and its target proteins in atherogenesis. Overall, the association between CHI3L1 and these target proteins in CVD suggests that CHI3L1 plays a critical role in CVD, including atherosclerosis, and that targeting CHI3L1 may be a promising therapeutic approach for atherosclerotic vascular disease.

## Involvement of CHI3L1 in autoimmune diseases

The development of other inflammatory diseases, such as rheumatoid arthritis (RA) and atopy, also involves the expression of CHI3L1. RA is an autoimmune disease that induces chronic inflammation of the joints. Several studies have suggested that CHI3L1 is a candidate autoantigen for inducing an autoimmune response in RA^184–189^. For example, patients with RA exhibited high levels of circulating CHI3L1, with high concentrations in synovial cells^190,191^. Moreover, cells that positively stained for major histocompatibility complex/human cartilage CHI3L1 complexes were observed in 61.5% of inflamed RA synovial samples compared with only 3.0% of the control samples in a specific and independent manner; therefore, CHI3L1 may be useful as a histological marker for the immunopathological diagnosis of RA^192^.

Articular chondrocytes, synovial cells, infiltrated macrophages, and neutrophils can produce CHI3L1 in RA-affected joints. In RA, levels of proinflammatory mediators (MMP-3, IL-6, IFN-γ, and TNF-α) correlate with CHI3L1, and anti-rheumatic factor therapy reduces CHI3L1 levels in patients^193–196^. The treatment of cartilage explants from young bovine stifle joints with IL-1β and TNF-α increased the release of CHI3L1 in association with an innate immune and stress response by chondrocytes, which may play a role in the host defense against pathogens or may protect cells against stress-induced damage^197^. In addition, miR-24 reduced the osteoblast apoptosis, abnormal bone formation, and mineralization induced by *Staphylococcus aureus* by inhibiting the expression of CHI3L1^198^. In turn, CHI3L1 was detected in RA synovial fluid and tissue from patients with arthritis, and the synovial fluid of three out of 10 patients with spondylarthritis exhibited endogenous CHI3L1 expression^186^. In a serum analysis of 25 patients with RA, we found that CHI3L1 levels in patients with RA were significantly higher than the normal level, and the elevated levels did not deviate among the patients. We also found that the receiver operating characteristic curve of CHI3L1 yielded an AUC value of 0.955, which is slightly lower than the AUC values of the US FDA-recommended RA diagnostic factors, i.e., CRP (0.988) and cyclic citrullinated peptide (CCP, 0.995), but slightly higher than that of rheumatoid factor (0.865).

There are a few reports of CHI3L1 as a target for the treatment of atopy. In fact, the serum levels of CHI3L1 were found to be significantly increased in patients with atopy compared with healthy controls^199^. A recent study indicated that CHI3L1 KO reduced allergic skin inflammation through the inhibition of Th2-mediated inflammation and M2 macrophage activation^102^. Moreover, the g.-247C/T polymorphism located in the *CHI3L1* promoter region is associated with the risk of atopy in Korean children^200^. Previously, we found that suppressing CHI3L1 alleviated atopic dermatitis-like skin inflammation by inhibiting NF-κB-mediated ITGA5 expression in CHI3L1 knockout mice^201^. In addition, treatment with a CHI3L1 siRNA reduced the levels of these inflammatory cytokines in TNF-α/IFN-γ-treated cells. Additionally, the administration of a commercially available anti-CHI3L1 antibody significantly alleviated atopic symptoms, reducing atopy-related cytokines and inflammatory cell recruitment. The AUC value for CHI3L1 (0.932) was significantly higher than that for IL-4 (0.650), IL-13 (0.785), and IL-1β (0.790) in the serum analysis of 20 atopy patients. DUPIXENT@ is the first FDA-approved biologic therapy targeting IL-4Rα, thereby inhibiting IL-4 and IL-13 signaling and reducing type 2 inflammation. Finally, we found that the CHI3L1-inhibiting Compound K284-6111 completely reduced atopy skin inflammation^202^. These findings suggest that CHI3L1 plays a role in the development of RA and atopy and is a good candidate therapeutic target for these inflammatory diseases.

## References

[1] Ochoa D (2021). Open targets platform: supporting systematic drug-target identification and prioritisation. Nucleic Acids Res..

[2] Koscielny G (2017). Open targets: a platform for therapeutic target identification and validation. Nucleic Acids Res..

[3] Carvalho-Silva D (2019). Open targets platform: new developments and updates two years on. Nucleic Acids Res..

[4] Snel B, Lehmann G, Bork P, Huynen MA (2000). STRING: a web-server to retrieve and display the repeatedly occurring neighbourhood of a gene. Nucleic Acids Res..

[5] von Mering C (2003). STRING: a database of predicted functional associations between proteins. Nucleic Acids Res..

[6] Szklarczyk D (2011). The STRING database in 2011: functional interaction networks of proteins, globally integrated and scored. Nucleic Acids Res..

[7] Szklarczyk D (2017). The STRING database in 2017: quality-controlled protein-protein association networks, made broadly accessible. Nucleic Acids Res..

[8] Szklarczyk D (2023). The STRING database in 2023: protein-protein association networks and functional enrichment analyses for any sequenced genome of interest. Nucleic Acids Res..

[9] Szklarczyk D (2021). The STRING database in 2021: customizable protein-protein networks, and functional characterization of user-uploaded gene/measurement sets. Nucleic Acids Res..

[10] Bussink AP, Speijer D, Aerts JM, Boot RG (2007). Evolution of mammalian chitinase(-like) members of family 18 glycosyl hydrolases. Genetics.

[11] Boot RG, Renkema GH, Strijland A, van Zonneveld AJ, Aerts JM (1995). Cloning of a cDNA encoding chitotriosidase, a human chitinase produced by macrophages. J. Biol. Chem..

[12] Bonneh-Barkay D (2012). Astrocyte and macrophage regulation of YKL-40 expression and cellular response in neuroinflammation. Brain Pathol..

[13] Hakala BE, White C, Recklies AD (1993). Human cartilage gp-39, a major secretory product of articular chondrocytes and synovial cells, is a mammalian member of a chitinase protein family. J. Biol. Chem..

[14] Henrissat B (1991). A classification of glycosyl hydrolases based on amino acid sequence similarities. Biochem. J..

[15] Kognole AA, Payne CM (2017). Inhibition of Mammalian Glycoprotein YKL-40: IDENTIFICATION OF THE PHYSIOLOGICAL LIGAND. J. Biol. Chem..

[16] Volck B (1998). YKL-40, a mammalian member of the chitinase family, is a matrix protein of specific granules in human neutrophils. Proc. Assoc. Am. Phys..

[17] Rehli M, Krause SW, Andreesen R (1997). Molecular characterization of the gene for human cartilage gp-39 (CHI3L1), a member of the chitinase protein family and marker for late stages of macrophage differentiation. Genomics.

[18] Hinsinger G (2015). Chitinase 3-like proteins as diagnostic and prognostic biomarkers of multiple sclerosis. Mult. Scler..

[19] Francescone RA (2011). Role of YKL-40 in the angiogenesis, radioresistance, and progression of glioblastoma. J. Biol. Chem..

[20] Faibish M, Francescone R, Bentley B, Yan W, Shao R (2011). A YKL-40-neutralizing antibody blocks tumor angiogenesis and progression: a potential therapeutic agent in cancers. Mol. Cancer Ther..

[21] Eurich K, Segawa M, Toei-Shimizu S, Mizoguchi E (2009). Potential role of chitinase 3-like-1 in inflammation-associated carcinogenic changes of epithelial cells. World J. Gastroenterol..

[22] Rathcke CN, Vestergaard H (2006). YKL-40, a new inflammatory marker with relation to insulin resistance and with a role in endothelial dysfunction and atherosclerosis. Inflamm. Res..

[23] Quintana E (2018). Cognitive impairment in early stages of multiple sclerosis is associated with high cerebrospinal fluid levels of chitinase 3-like 1 and neurofilament light chain. Eur. J. Neurol..

[24] Querol-Vilaseca M (2017). YKL-40 (Chitinase 3-like I) is expressed in a subset of astrocytes in Alzheimer’s disease and other tauopathies. J. Neuroinflamm..

[25] Kumagai E (2016). Serum YKL-40 as a marker of liver fibrosis in patients with non-alcoholic fatty liver disease. Sci. Rep..

[26] Di Rosa M, Szychlinska MA, Tibullo D, Malaguarnera L, Musumeci G (2014). Expression of CHI3L1 and CHIT1 in osteoarthritic rat cartilage model. A morphological study. Eur. J. Histochem..

[27] Di Rosa M, Malaguarnera L (2016). Chitinase 3 Like-1: an emerging molecule involved in diabetes and diabetic complications. Pathobiology.

[28] He CH (2013). Chitinase 3-like 1 regulates cellular and tissue responses via IL-13 receptor alpha2. Cell Rep..

[29] Subramaniam R, Mizoguchi A, Mizoguchi E (2016). Mechanistic roles of epithelial and immune cell signaling during the development of colitis-associated cancer. Cancer Res. Front..

[30] Low D (2015). Chitinase 3-like 1 induces survival and proliferation of intestinal epithelial cells during chronic inflammation and colitis-associated cancer by regulating S100A9. Oncotarget.

[31] Yu JE (2022). Anti-Chi3L1 antibody suppresses lung tumor growth and metastasis through inhibition of M2 polarization. Mol. Oncol..

[32] Ciledag A (2018). High serum YKL-40 level is associated with poor prognosis in patients with lung cancer. Tuberk Toraks.

[33] Hamilton G, Rath B, Burghuber O (2015). Chitinase-3-like-1/YKL-40 as marker of circulating tumor cells. Transl. Lung Cancer Res..

[34] Xu CH, Yu LK, Hao KK (2014). Serum YKL-40 level is associated with the chemotherapy response and prognosis of patients with small cell lung cancer. PLoS One.

[35] Kim HR (2012). Levels of YKL-40 in pleural effusions and blood from patients with pulmonary or pleural disease. Cytokine.

[36] Choi IK, Kim YH, Kim JS, Seo JH (2010). High serum YKL-40 is a poor prognostic marker in patients with advanced non-small cell lung cancer. Acta Oncol..

[37] Junker N, Johansen JS, Andersen CB, Kristjansen PE (2005). Expression of YKL-40 by peritumoral macrophages in human small cell lung cancer. Lung Cancer.

[38] Johansen JS, Drivsholm L, Price PA, Christensen IJ (2004). High serum YKL-40 level in patients with small cell lung cancer is related to early death. Lung Cancer.

[39] Wang J, Sheng Z, Yang W, Cai Y (2016). Elevated serum concentration of Chitinase 3-Like 1 is an independent prognostic biomarker for poor survival in lung cancer patients. Cell. Physiol. Biochem..

[40] Jefri M, Huang YN, Huang WC, Tai CS, Chen WL (2015). YKL-40 regulated epithelial-mesenchymal transition and migration/invasion enhancement in non-small cell lung cancer. BMC Cancer.

[41] Wang XW, Cai CL, Xu JM, Jin H, Xu ZY (2015). Increased expression of chitinase 3-like 1 is a prognosis marker for non-small cell lung cancer correlated with tumor angiogenesis. Tumour Biol..

[42] Rusak A, Jablonska K, Dziegiel P (2016). The role of YKL-40 in a cancerous process. Postepy Hig. Med. Dosw ..

[43] Thom I (2010). Elevated pretreatment serum concentration of YKL-40-An independent prognostic biomarker for poor survival in patients with metastatic nonsmall cell lung cancer. Cancer.

[44] Coffman FD (2008). Chitinase 3-Like-1 (CHI3L1): a putative disease marker at the interface of proteomics and glycomics. Crit. Rev. Clin. Lab. Sci..

[45] Johansen JS (2006). Studies on serum YKL-40 as a biomarker in diseases with inflammation, tissue remodelling, fibroses and cancer. Dan Med. Bull..

[46] Kim DH (2018). Regulation of chitinase-3-like-1 in T cell elicits Th1 and cytotoxic responses to inhibit lung metastasis. Nat. Commun..

[47] Corradi M (2013). YKL-40 and mesothelin in the blood of patients with malignant mesothelioma, lung cancer and asbestosis. Anticancer Res..

[48] Lee CM (2016). IL-13Ralpha2 uses TMEM219 in chitinase 3-like-1-induced signalling and effector responses. Nat .Commun..

[49] Lee CG (2009). Role of breast regression protein 39 (BRP-39)/chitinase 3-like-1 in Th2 and IL-13-induced tissue responses and apoptosis. J. Exp. Med..

[50] Yang PS (2022). Targeting protumor factor chitinase-3-like-1 secreted by Rab37 vesicles for cancer immunotherapy. Theranostics.

[51] Ma B (2022). CHI3L1 enhances melanoma lung metastasis via regulation of T cell co-stimulators and CTLA-4/B7 axis. Front. Immunol..

[52] Lee YS (2022). A small molecule targeting CHI3L1 inhibits lung metastasis by blocking IL-13Ralpha2-mediated JNK-AP-1 signals. Mol. Oncol..

[53] Hong DE (2022). A natural CHI3L1-targeting compound, ebractenoid F, inhibits lung cancer cell growth and migration and induces apoptosis by blocking CHI3L1/AKT signals. Molecules.

[54] Lauro S, Onesti CE, Righini R, Marchetti P (2014). The use of bevacizumab in non-small cell lung cancer: an update. Anticancer Res..

[55] Uprety D (2019). Clinical utility of ramucirumab in non-small-cell lung cancer. Biologics.

[56] Zhang Y (2016). Tumor-penetration and antitumor efficacy of cetuximab are enhanced by co-administered iRGD in a murine model of human NSCLC. Oncol. Lett..

[57] Agrawal S, Feng Y, Roy A, Kollia G, Lestini B (2016). Nivolumab dose selection: challenges, opportunities, and lessons learned for cancer immunotherapy. J. Immunother. Cancer.

[58] Low JL (2021). Low-dose pembrolizumab in the treatment of advanced non-small cell lung cancer. Int. J .Cancer.

[59] Bao J (2022). Serum CHI3L1 as a biomarker for non-invasive diagnosis of liver fibrosis. Discov. Med..

[60] Huang WS (2017). Correlation of Chitinase 3-Like 1 single nucleotide polymorphisms with hepatocellular carcinoma in Taiwan. Int. J. Med. Sci..

[61] Mangoud NOM, Ali SA, El Kassas M, Soror SH (2021). Chitinase 3-like-1, Tolloid-like protein 1, and intergenic gene polymorphisms are predictors for hepatocellular carcinoma development after hepatitis C virus eradication by direct-acting antivirals. IUBMB Life.

[62] Peltonen R (2020). Elevated serum YKL-40, IL-6, CRP, CEA, and CA19-9 combined as a prognostic biomarker panel after resection of colorectal liver metastases. PLoS One.

[63] Wang S (2022). Diagnostic and prognostic value of serum Chitinase 3-like protein 1 in hepatocellular carcinoma. J. Clin. Lab. Anal..

[64] Qiu QC (2018). CHI3L1 promotes tumor progression by activating TGF-beta signaling pathway in hepatocellular carcinoma. Sci. Rep..

[65] Lu D (2022). Multi-omics profiling reveals Chitinase-3-like protein 1 as a key mediator in the crosstalk between sarcopenia and liver cancer. Redox Biol..

[66] Shantha Kumara HM (2016). Plasma chitinase 3-like 1 is persistently elevated during first month after minimally invasive colorectal cancer resection. World J. Gastrointest. Oncol..

[67] Eldaly MN, Metwally FM, Shousha WG, El-Saiid AS, Ramadan SS (2020). Clinical potentials of miR-576-3p, miR-613, NDRG2 and YKL40 in colorectal cancer patients. Asian Pac. J. Cancer Prev..

[68] Kawada M (2012). Chitinase 3-like 1 promotes macrophage recruitment and angiogenesis in colorectal cancer. Oncogene.

[69] Watanabe K (2022). Chitinase 3-like 1 secreted from cancer-associated fibroblasts promotes tumor angiogenesis via interleukin-8 secretion in colorectal cancer. Int. J. Oncol..

[70] Liu K, Jin M, Ye S, Yan S (2020). CHI3L1 promotes proliferation and improves sensitivity to cetuximab in colon cancer cells by down-regulating p53. J. Clin. Lab. Anal..

[71] Yang MS (2008). Chitinase-3-like 1 (CHI3L1) gene and schizophrenia: genetic association and a potential functional mechanism. Biol. Psychiatry.

[72] Lananna BV (2020). Chi3l1/YKL-40 is controlled by the astrocyte circadian clock and regulates neuroinflammation and Alzheimer’s disease pathogenesis. Sci. Transl. Med..

[73] Craig-Schapiro R (2010). YKL-40: a novel prognostic fluid biomarker for preclinical Alzheimer’s disease. Biol. Psychiatry.

[74] Choi J, Lee H-W, Suk K (2011). Plasma level of chitinase 3-like 1 protein increases in patients with early Alzheimer’s disease. J. Neurol..

[75] Sanfilippo C, Malaguarnera L, Di Rosa M (2016). Chitinase expression in Alzheimer’s disease and non-demented brains regions. J. Neurol. Sci..

[76] Rakic S (2018). Systemic infection modifies the neuroinflammatory response in late stage Alzheimer’s disease. Acta Neuropathol. Commun..

[77] Sanfilippo C (2022). Sex-dependent neuro-deconvolution analysis of Alzheimer’s disease brain transcriptomes according to CHI3L1 expression levels. J. Neuroimmunol..

[78] Sanfilippo C (2017). CHI3L1 and CHI3L2 overexpression in motor cortex and spinal cord of sALS patients. Mol. Cell. Neurosci..

[79] Long X (2016). Hippocampal YKL-40 expression in rats after status epilepticus. Epilepsy Res..

[80] Carter SF (2019). Astrocyte biomarkers in Alzheimer’s disease. Trends Mol. Med..

[81] Teitsdottir UD (2021). Cerebrospinal fluid C18 ceramide associates with markers of Alzheimer’s disease and inflammation at the pre-and early stages of dementia. J. Alzheimers Dis..

[82] Watabe-Rudolph M (2012). Chitinase enzyme activity in CSF is a powerful biomarker of Alzheimer disease. Neurology.

[83] Groblewska M, Mroczko B (2017). YKL-40 as a potential biomarker and a possible target in therapeutic strategies of Alzheimer’s disease. Curr. Neuropharmacol..

[84] Bonneh‐Barkay D (2012). Astrocyte and macrophage regulation of YKL‐40 expression and cellular response in neuroinflammation. Brain Pathol..

[85] Moreno-Rodriguez M, Perez SE, Nadeem M, Malek-Ahmadi M, Mufson EJ (2020). Frontal cortex chitinase and pentraxin neuroinflammatory alterations during the progression of Alzheimer’s disease. J. Neuroinflamm..

[86] Naka KK (2018). Association of vascular indices with novel circulating biomarkers as prognostic factors for cardiovascular complications in patients with type 2 diabetes mellitus. Clin. Biochem..

[87] Molina-Martínez P (2021). Microglial hyperreactivity evolved to immunosuppression in the hippocampus of a mouse model of accelerated aging and Alzheimer’s Disease traits. Front. Aging Neurosci..

[88] Chen C-H (2012). Increased NF-κB signalling up-regulates BACE1 expression and its therapeutic potential in Alzheimer’s disease. Int. J. Neuropsychopharmacol..

[89] Rolova T (2016). Deletion of nuclear factor kappa B p50 subunit decreases inflammatory response and mildly protects neurons from transient forebrain ischemia-induced damage. Aging Dis..

[90] Snow WM, Albensi BC (2016). Neuronal gene targets of NF-κB and their dysregulation in Alzheimer’s disease. Front. Mol. Neurosci..

[91] Gispert JD (2017). The APOE ε4 genotype modulates CSF YKL-40 levels and their structural brain correlates in the continuum of Alzheimer’s disease but not those of sTREM2. Alzheimers Dement.

[92] Zhang H (2018). Cerebrospinal fluid phosphorylated tau, visinin-like protein-1, and chitinase-3-like protein 1 in mild cognitive impairment and Alzheimer’s disease. Alzheimers Dement.

[93] Llorens F (2017). YKL-40 in the brain and cerebrospinal fluid of neurodegenerative dementias. Int. J. Mol. Sci..

[94] Jung YY (2018). Atherosclerosis is exacerbated by chitinase-3-like-1 in amyloid precursor protein transgenic mice. Theranostics.

[95] Choi JY (2018). K284-6111 prevents the amyloid beta-induced neuroinflammation and impairment of recognition memory through inhibition of NF-κB-mediated CHI3L1 expression. J. Neuroinflamm..

[96] Ham HJ (2020). K284-6111 alleviates memory impairment and neuroinflammation in Tg2576 mice by inhibition of Chitinase-3-like 1 regulating ERK-dependent PTX3 pathway. J. Neuroinflamm..

[97] Chung C, Tallerico T, Seeman P (2003). Schizophrenia hippocampus has elevated expression of chondrex glycoprotein gene. Synapse.

[98] Zhao X (2007). Functional variants in the promoter region of chitinase 3–like 1 (CHI3L1) and susceptibility to schizophrenia. Am. J. Hum. Genet..

[99] Ohi K (2010). The chitinase 3-like 1 gene and schizophrenia: Evidence from a multi-center case–control study and meta-analysis. Schizophr. Res..

[100] Gomez JL (2015). Genetic variation in chitinase 3-like 1 (CHI3L1) contributes to asthma severity and airway expression of YKL-40. J. Allergy Clin. Immunol..

[101] Yamada K (2009). Failure to confirm genetic association of the CHI3L1 gene with schizophrenia in Japanese and Chinese populations. Am. J. Med. Genet. B Neuropsychiatr. Genet..

[102] Kwak EJ (2019). Chitinase 3-like 1 drives allergic skin inflammation via Th2 immunity and M2 macrophage activation. Clin. Exp. Allergy.

[103] Libreros S (2015). Allergen induced pulmonary inflammation enhances mammary tumor growth and metastasis: Role of CHI3L1. J. Leukoc. Biol..

[104] Müller N (2012). Impaired monocyte activation in schizophrenia. Front. Neurosci..

[105] Khandaker GM, Dantzer R (2016). Is there a role for immune-to-brain communication in schizophrenia?. Psychopharmacology.

[106] Upthegrove R, Khandaker GM (2020). Cytokines, oxidative stress and cellular markers of inflammation in schizophrenia. Curr. Top Behav. Neurosci..

[107] Emamian ES, Hall D, Birnbaum MJ, Karayiorgou M, Gogos JA (2004). Convergent evidence for impaired AKT1-GSK3β signaling in schizophrenia. Nat. Genet..

[108] Zhao T, Su Z, Li Y, Zhang X, You Q (2020). Chitinase-3 like-protein-1 function and its role in diseases. Signal. Transduct. Target Ther..

[109] Lencz T (2007). Converging evidence for a pseudoautosomal cytokine receptor gene locus in schizophrenia. Mol. Psychiatry.

[110] Arion D, Unger T, Lewis DA, Levitt P, Mirnics K (2007). Molecular evidence for increased expression of genes related to immune and chaperone function in the prefrontal cortex in schizophrenia. Biol. Psychiatry.

[111] Hwang Y (2013). Gene expression profiling by mRNA sequencing reveals increased expression of immune/inflammation-related genes in the hippocampus of individuals with schizophrenia. Transl. Psychiatry.

[112] Orhan F (2018). Increased number of monocytes and plasma levels of MCP‐1 and YKL‐40 in first‐episode psychosis. Acta Psychiatr. Scand..

[113] Yeo IJ (2023). Overexpression of transmembrane TNFalpha in brain endothelial cells induces schizophrenia-relevant behaviors. Mol. Psychiatry.

[114] Piñero J (2017). DisGeNET: a comprehensive platform integrating information on human disease-associated genes and variants. Nucleic Acids Res..

[115] Hall S (2018). Cerebrospinal fluid concentrations of inflammatory markers in Parkinson’s disease and atypical parkinsonian disorders. Sci. Rep..

[116] Aarsland D (2021). Parkinson disease-associated cognitive impairment. Nat. Rev. Dis. Primers.

[117] Anwar MM, Fathi MH (2023). Early approaches of YKL-40 as a biomarker and therapeutic target for Parkinson’s disease. Neurodegener Dis. Manag..

[118] Vu L (2020). Cross-sectional and longitudinal measures of chitinase proteins in amyotrophic lateral sclerosis and expression of CHI3L1 in activated astrocytes. J. Neurol. Neurosurg. Psychiatry.

[119] Vinther-Jensen T (2016). Selected CSF biomarkers indicate no evidence of early neuroinflammation in Huntington disease. Neurol. Neuroimmunol. Neuroinflamm..

[120] Quintana E (2018). Cognitive impairment in early stages of multiple sclerosis is associated with high cerebrospinal fluid levels of chitinase 3‐like 1 and neurofilament light chain. Eur. J. Neurol..

[121] Floro S (2022). Role of Chitinase 3–like 1 as a biomarker in multiple sclerosis: a systematic review and meta-analysis. Neurol. Neuroimmunol. Neuroinflamm..

[122] Cong S, Xiang C, Wang H, Cong S (2021). Diagnostic utility of fluid biomarkers in multiple system atrophy: a systematic review and meta-analysis. J. Neurol..

[123] Autar K (2021). ASNTR Abstracts 2021. Cell Transplant.

[124] Langenbruch L, Wiendl H, Groß C, Kovac S (2021). Diagnostic utility of cerebrospinal fluid (CSF) findings in seizures and epilepsy with and without autoimmune-associated disease. Seizure.

[125] Gimbrone MA, Garcia-Cardena G (2016). Endothelial cell dysfunction and the pathobiology of atherosclerosis. Circ. Res..

[126] Basatemur GL, Jorgensen HF, Clarke MCH, Bennett MR, Mallat Z (2019). Vascular smooth muscle cells in atherosclerosis. Nat. Rev. Cardiol..

[127] Dong Y, Zhang Y, Yang X, Yan C, Feng Y (2022). Recent insights into neutrophil extracellular traps in cardiovascular diseases. J. Clin. Med..

[128] Moroni F, Ammirati E, Norata GD, Magnoni M, Camici PG (2019). The role of monocytes and macrophages in human atherosclerosis, plaque neoangiogenesis, and atherothrombosis. Mediators Inflamm..

[129] Boot RG (1999). Strong induction of members of the chitinase family of proteins in atherosclerosis: chitotriosidase and human cartilage gp-39 expressed in lesion macrophages. Arterioscler Thromb Vasc. Biol..

[130] Xu T (2016). YKL-40 level and hypertension incidence: a population-based nested case-control study in China. J. Am. Heart Assoc..

[131] Malyszko J, Koc-Zorawska E, Malyszko J (2014). YKL-40, a marker of cardiovascular disease and endothelial dysfunction, in kidney transplant recipients. Transplant Proc..

[132] Erfan G (2015). Serum YKL-40: a potential biomarker for psoriasis or endothelial dysfunction in psoriasis?. Mol. Cell. Biochem..

[133] Jafari B, Mohsenin V (2016). Chitinase-3-like protein-1 (YKL-40) as a marker of endothelial dysfunction in obstructive sleep apnea. Sleep Med..

[134] Keskin GS (2019). Relationship between plasma YKL-40 levels and endothelial dysfunction in chronic kidney disease. Turk. J. Med. Sci..

[135] Kocyigit I (2015). The serum YKL-40 level is associated with vascular injury and predicts proteinuria in nephrotic syndrome patients. J. Atheroscler. Thromb..

[136] Zheng JL (2010). Increased serum YKL-40 and C-reactive protein levels are associated with angiographic lesion progression in patients with coronary artery disease. Atherosclerosis.

[137] Michelsen AE (2010). Increased YKL-40 expression in patients with carotid atherosclerosis. Atherosclerosis.

[138] Jiao Y (2020). Early identification of carotid vulnerable plaque in asymptomatic patients. BMC Cardiovasc. Disord..

[139] Wu S (2014). Circulating YKL-40 level, but not CHI3L1 gene variants, is associated with atherosclerosis-related quantitative traits and the risk of peripheral artery disease. Int. J. Mol. Sci..

[140] Hobaus C (2018). YKL-40 levels increase with declining ankle-brachial index and are associated with long-term cardiovascular mortality in peripheral arterial disease patients. Atherosclerosis.

[141] Aguilera E (2015). Relationship of YKL-40 and adiponectin and subclinical atherosclerosis in asymptomatic patients with type 1 diabetes mellitus from a European Mediterranean population. Cardiovasc. Diabetol..

[142] Bakirci EM (2015). Serum YKL-40/chitinase 3-like protein 1 level is an independent predictor of atherosclerosis development in patients with obstructive sleep apnea syndrome. Turk Kardiyol Dern. Ars..

[143] Kulkarni NB, Ganu MU, Godbole SG, Deo SS (2018). Assessment of potential biomarkers of atherosclerosis in Indian patients with type 2 diabetes mellitus. Indian J. Med. Res..

[144] Kwon Y (2020). Serum YKL-40 levels are associated with the atherogenic index of plasma in children. Mediators Inflamm..

[145] Yamada K, Hyodo T, Urabe S, Haga S, Hosaka T (2022). Serum YKL-40 level is associated with Geriatric Nutritional Risk Index (GNRI) and γ-GTP in hemodialysis patients. J. Med. Investig..

[146] Chen XL (2017). Serum YKL-40, a prognostic marker in patients with large-artery atherosclerotic stroke. Acta Neurol. Scand..

[147] Akboga MK, Yalcin R, Sahinarslan A, Yilmaz Demirtas C, Abaci A (2016). Effect of serum YKL-40 on coronary collateral development and SYNTAX score in stable coronary artery disease. Int. J. Cardiol..

[148] Sciborski K (2018). Plasma YKL-40 levels correlate with the severity of coronary atherosclerosis assessed with the SYNTAX score.. Pol. Arch. Intern. Med..

[149] Schroder J (2020). Prognosis and reclassification by YKL-40 in stable coronary artery disease. J. Am. Heart Assoc..

[150] Przybyłowski P (2014). YKL-40, a novel marker of cardiovascular complications, is related to kidney function in heart transplant recipients. Transplant Proc..

[151] Laucyte-Cibulskiene A (2021). Role of GDF-15, YKL-40 and MMP 9 in patients with end-stage kidney disease: focus on sex-specific associations with vascular outcomes and all-cause mortality. Biol. Sex Differ..

[152] Chen G (2014). Elevated plasma YKL-40 as a prognostic indicator in patients with idiopathic pulmonary arterial hypertension. Respirology.

[153] Masajtis-Zagajewska A, Majer J, Nowicki M (2010). Effect of moxonidine and amlodipine on serum YKL-40, plasma lipids and insulin sensitivity in insulin-resistant hypertensive patients-a randomized, crossover trial. Hypertens. Res..

[154] Mathiasen AB (2011). Plasma YKL-40 in relation to the degree of coronary artery disease in patients with stable ischemic heart disease. Scand. J. Clin. Lab. Investig..

[155] Qi R (2019). Effect of laparoscopic splenectomy on portal vein thrombosis and serum YKL-40 in patients with cirrhotic portal hypertension. Ann. Hepatol..

[156] Sun X (2022). Chitinase 3 like 1 contributes to the development of pulmonary vascular remodeling in pulmonary hypertension. JCI Insight.

[157] Xing Y, Guo J, Gai L, Liu B, Luo D (2020). Serum YKL-40 is associated with the severity of coronary artery disease and hypertension. Asian J. Surg..

[158] Xu T (2017). YKL-40 is a novel biomarker for predicting hypertension incidence among prehypertensive subjects: a population-based nested case-control study in China. Clin. Chim. Acta.

[159] Cetin M (2020). Elevated serum YKL40 level is a predictor of MACE during the long-term follow up in hypertensive patients. Clin. Exp. Hypertens..

[160] Li K, Chen Z, Qin Y, Wei YX (2019). Plasm YKL-40 levels are associated with hypertension in patients with obstructive sleep apnea. Biomed. Res. Int..

[161] Karalilova R (2019). Serum YKL-40 and IL-6 levels correlate with ultrasound findings of articular and periarticular involvement in patients with systemic sclerosis. Rheumatol. Int..

[162] Nordenbaek C (2005). High serum levels of YKL-40 in patients with systemic sclerosis are associated with pulmonary involvement. Scand. J. Rheumatol..

[163] Furukawa T (2019). Relationship between YKL-40 and pulmonary arterial hypertension in systemic sclerosis. Mod. Rheumatol..

[164] Konig K (2016). BNP, troponin I, and YKL-40 as screening markers in extremely preterm infants at risk for pulmonary hypertension associated with bronchopulmonary dysplasia. Am. J. Physiol. Lung Cell. Mol. Physiol..

[165] Zhou Y, Meng LJ, Wang J (2020). Changes in serum human cartilage glycoprotein-39 and high-mobility group box 1 in preterm infants with bronchopulmonary dysplasia. Zhongguo Dang Dai Er Ke Za Zhi.

[166] Bilim O (2010). Serum YKL-40 predicts adverse clinical outcomes in patients with chronic heart failure. J. Card Fail.

[167] Ma WH (2012). Association between human cartilage glycoprotein 39 (YKL-40) and arterial stiffness in essential hypertension. BMC Cardiovasc. Disord..

[168] Ma CY (2018). Change of inflammatory factors in patients with acute coronary syndrome. Chin Med. J..

[169] Harutyunyan M (2012). The inflammatory biomarker YKL-40 as a new prognostic marker for all-cause mortality in patients with heart failure. Immunobiology.

[170] Arain F (2020). YKL-40 (Chitinase-3-Like Protein 1) Serum Levels in Aortic Stenosis. Circ. Heart Fail.

[171] Mathiasen AB, Henningsen KM, Harutyunyan MJ, Mygind ND, Kastrup J (2010). YKL-40: a new biomarker in cardiovascular disease?. Biomark. Med..

[172] Bakirci EM (2015). New inflammatory markers for prediction of non-dipper blood pressure pattern in patients with essential hypertension: serum YKL-40/Chitinase 3-like protein 1 levels and echocardiographic epicardial adipose tissue thickness. Clin. Exp. Hypertens..

[173] Vergadi E (2011). Early macrophage recruitment and alternative activation are critical for the later development of hypoxia-induced pulmonary hypertension. Circulation.

[174] Waki H, Gouraud SS, Maeda M, Paton JF (2008). Gene expression profiles of major cytokines in the nucleus tractus solitarii of the spontaneously hypertensive rat. Auton. Neurosci..

[175] Ross R (1993). The pathogenesis of atherosclerosis: a perspective for the 1990s. Nature.

[176] Libby P, Ridker PM, Maseri A (2002). Inflammation and atherosclerosis. Circulation.

[177] Cines DB (1998). Endothelial cells in physiology and in the pathophysiology of vascular disorders. Blood.

[178] Galley HF, Webster NR (2004). Physiology of the endothelium. Br. J. Anaesth..

[179] Sima AV, Stancu CS, Simionescu M (2009). Vascular endothelium in atherosclerosis. Cell Tissue Res..

[180] Deanfield JE, Halcox JP, Rabelink TJ (2007). Endothelial function and dysfunction: testing and clinical relevance. Circulation.

[181] Shackelton LM, Mann DM, Millis AJ (1995). Identification of a 38-kDa heparin-binding glycoprotein (gp38k) in differentiating vascular smooth muscle cells as a member of a group of proteins associated with tissue remodeling. J. Biol. Chem..

[182] Malinda KM, Ponce L, Kleinman HK, Shackelton LM, Millis AJ (1999). Gp38k, a protein synthesized by vascular smooth muscle cells, stimulates directional migration of human umbilical vein endothelial cells. Exp. Cell. Res..

[183] Jung TW (2018). Chitinase-3-like protein 1 ameliorates atherosclerotic responses via PPARδ-mediated suppression of inflammation and ER stress. J. Cell. Biochem..

[184] Steenbakkers PG (2003). Localization of MHC class II/human cartilage glycoprotein-39 complexes in synovia of rheumatoid arthritis patients using complex-specific monoclonal antibodies. J. Immunol..

[185] van Bilsen JH (2004). Functional regulatory immune responses against human cartilage glycoprotein-39 in health vs. proinflammatory responses in rheumatoid arthritis. Proc. Natl. Acad. Sci. USA.

[186] van Lierop MJ (2007). Endogenous HLA-DR-restricted presentation of the cartilage antigens human cartilage gp-39 and melanoma inhibitory activity in the inflamed rheumatoid joint. Arthritis. Rheum..

[187] Vos K (2000). Raised human cartilage glycoprotein-39 plasma levels in patients with rheumatoid arthritis and other inflammatory conditions. Ann. Rheum. Dis.

[188] Tsark EC (2002). Differential MHC class II-mediated presentation of rheumatoid arthritis autoantigens by human dendritic cells and macrophages. J. Immunol..

[189] Vos K (2000). Cellular immune response to human cartilage glycoprotein-39 (HC gp-39)-derived peptides in rheumatoid arthritis and other inflammatory conditions. Rheumatology.

[190] Johansen JS, Jensen HS, Price PA (1993). A new biochemical marker for joint injury. Analysis of YKL-40 in serum and synovial fluid. Br. J. Rheumatol..

[191] Volck B (2001). Studies on YKL-40 in knee joints of patients with rheumatoid arthritis and osteoarthritis. Involvement of YKL-40 in the joint pathology. Osteoarthritis Cartilage.

[192] Baeten D (2004). Detection of major histocompatibility complex/human cartilage gp-39 complexes in rheumatoid arthritis synovitis as a specific and independent histologic marker. Arthritis Rheum..

[193] Cope AP (1999). T cell responses to a human cartilage autoantigen in the context of rheumatoid arthritis-associated and nonassociated HLA-DR4 alleles. Arthritis Rheum..

[194] Vaananen T (2017). Glycoprotein YKL-40: A potential biomarker of disease activity in rheumatoid arthritis during intensive treatment with csDMARDs and infliximab. Evidence from the randomised controlled NEO-RACo trial. PLoS One.

[195] Houseman M (2012). Baseline serum MMP-3 levels in patients with Rheumatoid Arthritis are still independently predictive of radiographic progression in a longitudinal observational cohort at 8 years follow up. Arthritis Res. Ther..

[196] Mottonen T (1999). Comparison of combination therapy with single-drug therapy in early rheumatoid arthritis: a randomised trial. FIN-RACo trial group. Lancet.

[197] Stevens AL, Wishnok JS, Chai DH, Grodzinsky AJ, Tannenbaum SR (2008). A sodium dodecyl sulfate-polyacrylamide gel electrophoresis-liquid chromatography tandem mass spectrometry analysis of bovine cartilage tissue response to mechanical compression injury and the inflammatory cytokines tumor necrosis factor alpha and interleukin-1beta. Arthritis Rheum..

[198] Jin T (2015). The Role of MicroRNA, miR-24, and Its Target CHI3L1 in Osteomyelitis Caused by Staphylococcus aureus. J. Cell. Biochem..

[199] Salomon J, Matusiak L, Nowicka-Suszko D, Szepietowski JC (2017). Chitinase-3-Like Protein 1 (YKL-40) Reflects the Severity of Symptoms in Atopic Dermatitis. J. Immunol. Res..

[200] Sohn MH (2009). Genetic variation in the promoter region of chitinase 3-like 1 is associated with atopy. Am. J .Respir. Crit. Care Med..

[201] Lee YS (2022). New therapeutic strategy for atopic dermatitis by targeting CHI3L1/ITGA5 axis. Clin. Transl. Med..

[202] Jeon SH (2021). Inhibition of Chitinase-3-like-1 by K284-6111 Reduces Atopic Skin Inflammation via Repressing Lactoferrin. Immune Netw..

